# Zinc Intakes and Health Outcomes: An Umbrella Review

**DOI:** 10.3389/fnut.2022.798078

**Published:** 2022-02-08

**Authors:** Jin Li, Dehong Cao, Yin Huang, Bo Chen, Zeyu Chen, Ruyi Wang, Qiang Dong, Qiang Wei, Liangren Liu

**Affiliations:** ^1^Department of Urology, Institute of Urology, National Clinical Research Center for Geriatrics, West China Hospital, Sichuan University, Chengdu, China; ^2^West China School of Medicine, Sichuan University, Chengdu, China

**Keywords:** dietary zinc, meta-analysis, supplementation, supplementary zinc, umbrella review

## Abstract

It is widely accepted that the zinc element is crucial in human beings. Zinc has gained more attention during the COVID-19 pandemic due to its utilization for the treatment and prevention of respiratory tract infections. However, some studies also pointed out that zinc intake might cause unwanted side effects and even be dangerous when overdosed. To reveal the relationship between zinc intake and health outcomes, we performed an umbrella review from human studies. In total, the umbrella review included 43 articles and identified 11 outcomes for dietary zinc intake and 86 outcomes for supplementary zinc intake. Dietary zinc intake in the highest dose would decrease the risk of overall and specific digestive tract cancers, depression, and type 2 diabetes mellitus (T2DM) in adults. Supplementary zinc consumption in adults was linked to an improvement of depression, antioxidant capacity and sperm quality, higher serum zinc concentration, and lower concentration of inflammatory markers. Zinc supplementation in children would reduce the incidence of diarrhea and pneumonia, improve zinc deficiency and boost growth. However, zinc might not decrease all-cause mortality in adults or the in-hospital mortality of COVID-19. And better maternal and neonatal outcomes may not derive from pregnant women who consumed higher or lower doses of zinc supplementation (>20 mg/day and <20 mg/day, respectively). Dose-response analyses revealed that a daily 5 mg increment of zinc would lower the risk of colorectal and esophageal cancer, whereas a large dose of zinc supplementation (daily 100 mg) showed no benefit in reducing prostate cancer risk.

## Introduction

As one of the trace elements, zinc plays an indispensable part in multiple metabolic processes from protein synthesis to immunity construction to gene expression Shankar, Prasad (1, 2). Zinc is comprised of over 1000 transcription factors and functions as a structural and regulatory component of over 300 enzymes *in vivo* (3). Hence, it is imaginable that zinc deficiency could result in a wide range of disorders in the human body. Since its first discovery in 1963, people who suffered from zinc deficiency in various severities would have symptoms including diarrhea (4), compromised immune function (5), infections (1), loss of memory (6), cognitive disorders (7), sperm damage in males and etc (1). From another aspect, zinc deficiency was gradually thought to be the risk factor of anemia (8), cognitive disorders (8), gastrointestinal dysfunction (1), hepatosplenomegaly (1), hypogonadism (8, 9), and so on. Furthermore, zinc therapy is to some certain extent applied in clinical treatment and prevention of COVID-19 (10), which further proved the importance of zinc.

**Figure 1:**
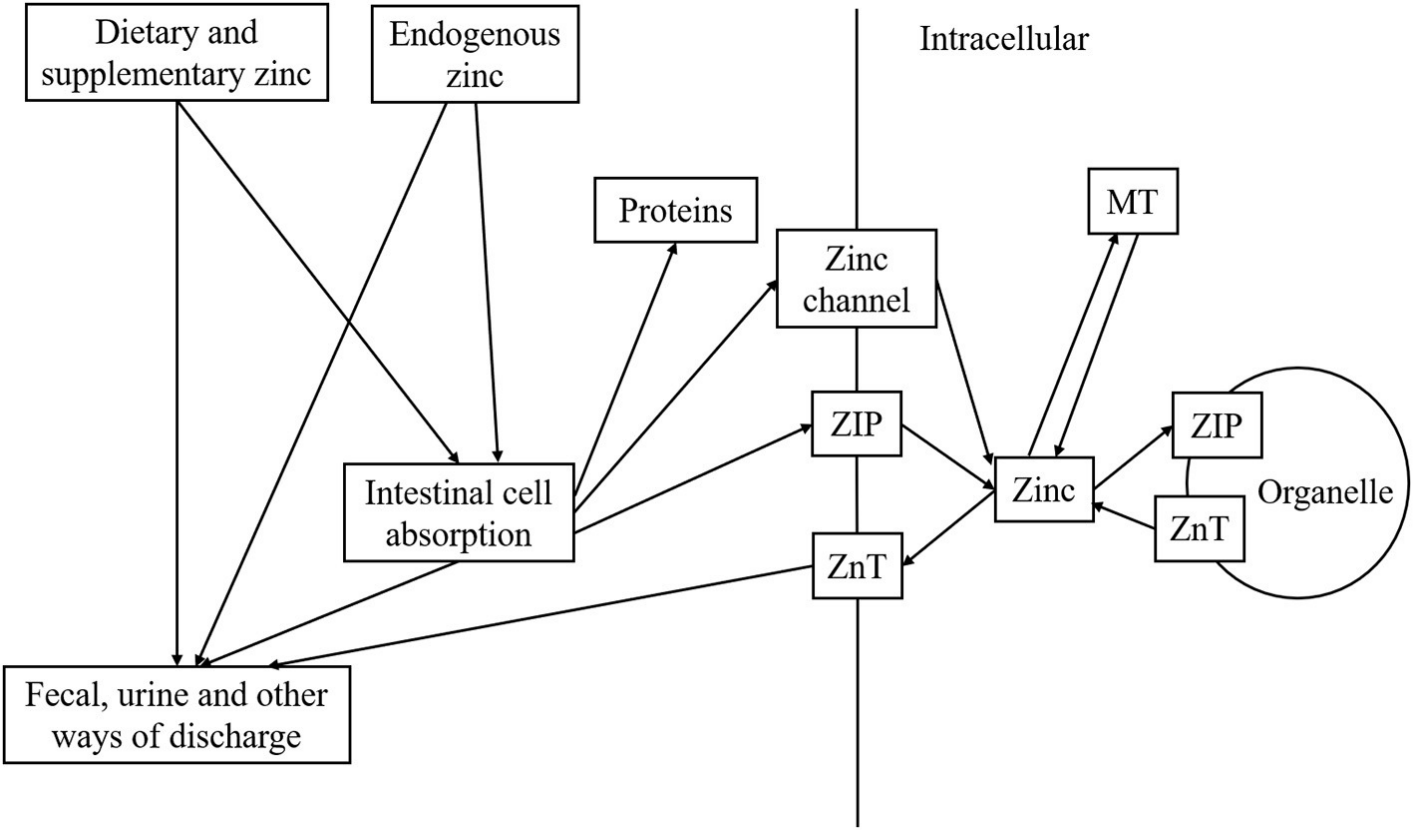
Metabolism of zinc in human body.

Zinc is distributed in a wide range of food, including meat (fish, red meat, and meat products), grains, cereals, dairy products, and dietary supplements (11). Apart from exogenous zinc, there might be several potential endogenous zinc-preserving organs: pancreas, hepatobiliary, gastroduodenal epithelium, and other possible sites (12). The exogenous zinc and endogenously secreted zinc enter the proximal small bowel, the primary zinc absorption place, and are absorbed into the basolateral membrane and transported into cellular organelle or portal circulation (1, 12). At the whole-body level, the discharge of zinc elements is primarily maintained by excretion from feces and urine (13). Intracellularly, zinc homeostasis is achieved by zinc transporters (ZnT), zinc-iron permeases (ZIP), and by metallothioneins (MT) (14). ZIP, also knowns as SLC39A, transports zinc from the extracellular matrix and intracellular vesicles to intracellular cytosol; ZnT, also known as SLC30A, is responsible for moving zinc from the intracellular matrix into extracellular space or intracellular zinc-preserving organelles (3, 5, 12). Zinc binding sites of MTs bind to zinc ions under the impact of signals, resulting in a flexible zinc concentration intracellularly (3, 5, 12). Zinc metabolism then lends itself to reach homeostasis appropriately to meet all required biological activities (Figure 1).

Until now, the associations between human zinc intakes and a wide range of health outcomes have been explored extensively. Nevertheless, there have been inconsistent conclusions about some specific outcomes, for instance, acute respiratory tract infection (including COVID-19) (10, 15–20). Besides, the effect of zinc can be dangerous sometimes. Excess consumption of zinc could exert the immunosuppressive effect to promote the multiplication of pathogens, which could be alerted upon intake (5). Therefore, a comprehensive systematic review is coined to summarize what is known and unknown about the effect of zinc, which gives inspirations for clinical and scientific practice. As such, we conducted an umbrella review to comprehensively summarize all available evidence of the effect of zinc intakes (including dietary and supplementary) in the human population.

## Method Section

### Umbrella Review and Literature Search

Umbrella review is characterized as an integrative review of eligible meta-analyses and (or) systematic reviews, designated to provide a broader overview about the related topic. It helps examine the aspects of the zinc effect which are most-heated explored as well as potentially underexplored, and then to propose recommendations for practice and research. We systematically searched, retracted, and organized the available data related to dietary and supplementary zinc consumption and multiple health outcomes. The intakes of zinc are usually measured by the form of zinc gluconate, sulfate, and acetate, making itself easily be calculated by specific zinc element dose per day or week, therefore systematic reviews without meta-analyses are excluded in our review.

Until December 2021, two authors (JL and DHC) systematically searched four electronic databases for articles that explored the associations between zinc intake and different health outcomes: PubMed, Embase, Web of Science, and the Cochrane Database of Systematic Reviews. The following search terms were used: (zinc OR zinc intake^*^ OR zinc consumption^*^) AND (systematic review^*^ OR meta-analys^*^), using truncated terminology following the SIGN guidance (21). Additionally, the lists of references of eligible literature were also manually screened for inclusion. Any discrepancies would be resolved by discussion or consultation with a third author.

### Eligibility Criteria

Articles with meta-analyses were considered eligible if (1) interventions were dietary or supplementary zinc intake and outcomes were the health outcomes, (2) participants were human regardless of age, sex, ethnicity, and country, (3) study designs were interventional studies (randomized controlled trial (RCT) or observational (case-control, cross-sectional, cohort studies), (4) metrics of the studies were effect size (ES), mean difference (MD), odds ratio (OR), relative risk (RR), standardized mean difference (SMD) or weighted mean difference (WMD). Articles were excluded if they (1) were systematic reviews without meta-analyses, (2) were animal studies or *in vitro*, (3) focused on the therapeutic aspect of zinc, (4) were published in languages other than English, (5) used undefined methodology. Whenever an article presented two or more health outcomes or displayed in different clinical settings, they were then extracted separately. When more than one study reported data for the same outcome, then we would select the most recent one with the largest sample size.

### Data Extraction

Two authors (JL and DHC) independently extracted the following data from eligible studies: (1) first author and publication year, (2) population, (3) the number of cases and participants in each study, (4) category of exposure (dietary and supplementary zinc), (5) outcome, (6) the estimated summary effect (ES, MD, OR, RR, SMD, and WMD), (7) corresponding 95% confidence intervals (CIs), (8) the number of included studies, (9) study design (cohort, case-control, cross-sectional and RCT), (10) type of comparisons (<20 mg/day Vs. never, >20 mg/day vs. never, highest vs. lowest and increment of 5 mg or 100 mg of dietary or supplementary zinc), (11) effect model (fixed or random), (12) *I*^2^ statistic value, (13) Cochran's Q test *P*-value, (14) Egger's test *P* value. If more than one outcome was reported in one article, we extracted each outcome respectively. In addition, supplementary zinc intakes described in each article were transformed into elemental zinc doses for better comparison. Any discrepancies would be resolved by consensus or consultation with a third author, who made the final decision.

### Quality Assessment and Evidence Grading

AMSTAR2, containing a comprehensive rating than the original AMSTAR classification, was utilized to evaluate the methodological quality and risk of biases of included articles (22–24). AMSTAR2 consisted of 16 items including 7 critical and 9 non-critical domains and classified different articles into “Critically low,” “Low,” “Moderate” and “High.” We assessed the strength of evidence of eligible articles through the Grading of Recommendations, Assessment, Development and Evaluation (GRADE) (25). All articles were categorized into four levels: “Very low,” “Low,” “Moderate” and “High.”

### Data Analysis

We retrieved the outcome data and the most adjusted estimated effect with 95% CI in each meta-analysis either through fixed or random effect. Dose-response calculations were extracted when available. When an article reported summary estimate effects of cohort and case-control studies separately without an overall outcome, the cohort studies were included in this review because they were generally less susceptible to selection and recall biases. We included heterogeneity, represented by *I*^2^ metric value and Cochran's *Q* test *P*-value. And publication bias was calculated by Egger's regression test (26). The standard that *P*-value < 0.05 was set for both Egger's test and heterogeneity.

## Results

### Characteristics of the Included Studies

**Figure 2:**
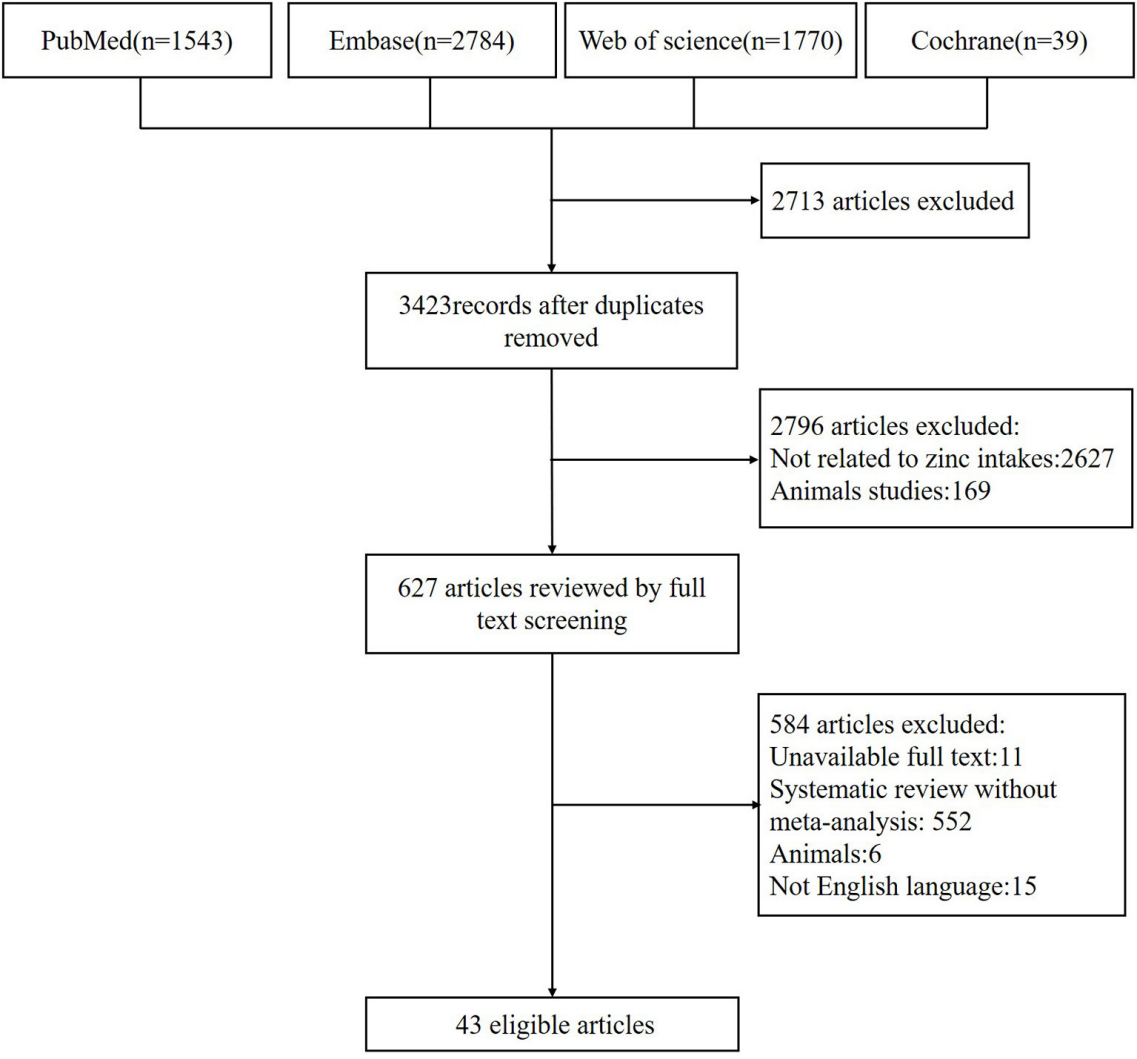
Flowchart of the study selection.

**Figure 3:**
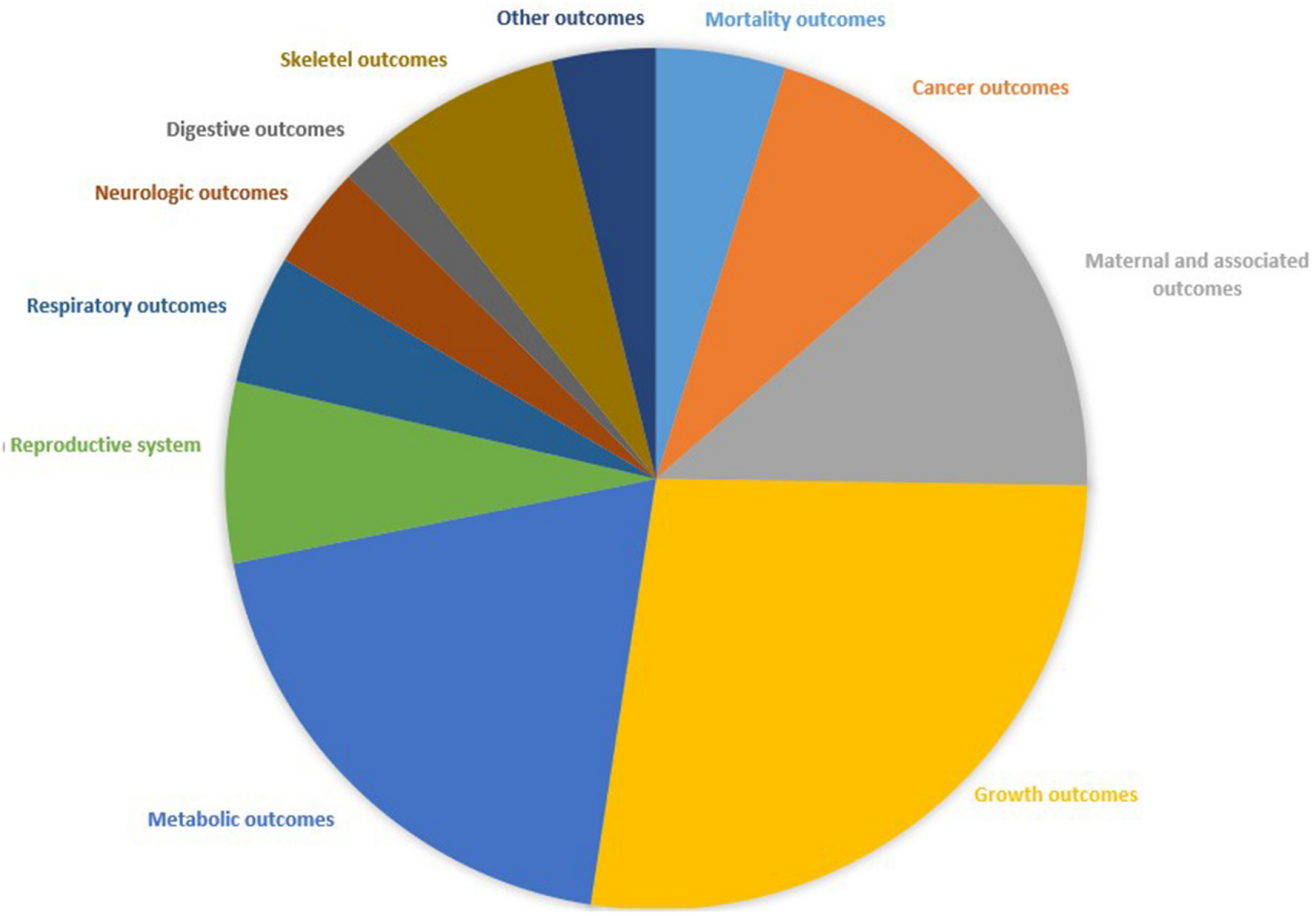
Map of health outcomes related to zinc intakes.

Figure 2 shows the whole process of systematic search and selection of eligible studies. The search identified a total of 6136 articles and yielded 43 meta-analyses for the umbrella review. And among them, we retrieved 11 unique outcomes for dietary zinc consumption and 88 unique outcomes for supplementary zinc intakes (Figure 3). The characteristics of the included studies as to dietary and supplementary zinc consumption were displayed in Table 1 and Supplementary Tables 1, 2.

**Table 1:** Associations between zinc intakes and mortality and cancer outcomes.

**Outcome**	**Author-Year**	**Type**	**Population**	**No. of cases/total**	**Metrics**	**Estimates**	**95%CI**	**No. of studies**	**Cohort**	**Case control**	**Cross-sectional**	**RCT**	**Effects model**	**I^**2**^**	**Q test p value**	**Egger** **test** ***p* value**
**Mortality outcomes**																
*Insignificant associations*																
All-cause mortality	Jayedi 2018	Diet	Adults	1,220/11,353	RR^a^	0.90	0.63, 1.16	3	3	0	0	0	Random	48	0.14	NA
All-cause mortality	Kanellopoulou 2021	Supplement	Adults	NA/4,382	RR^b^	0.90	0.69, 1.17	2	2	0	0	0	Random	0	0.511	NA
All-cause mortality	Tam 2020	Supplement	Children	NA/76,900	RR^b^	0.24	0.04, 1.62	3	0	0	0	3	Random	100	<0.002	NA
**Cancer outcomes**																
*Significant associations*																
Colorectal cancer	Qiao 2013	Diet	Adults	5,676/350,307	RR^c^	0.86	0.78, 0.96	6	6	0	0	0	Random	33.5	0.16	No
Esophageal cancer	Ma 2018	Diet	Adults	1,513/51,628	OR^c^	0.85	0.77, 0.93	5	1	4	0	0	Random	54.2	0.068	No
Digestive tract cancers	Li 2014	Diet	Adults	10,675/395,222	RR^a^	0.82	0.70, 0.96	19	6	13	0	0	Random	75.7	<0.001	No
Colorectal cancer	Li 2014	Diet	Adults	NA/35,2319	RR^a^	0.80	0.69, 0.92	6	5	1	0	0	Random	60.5	0.027	NA
Pancreatic cancer	Li 2017	Diet	Adults	1,659/106,359	RR^a^	0.798	0.621, 0.984	7	2	5	0	0	Random	58.2	0.026	0.997
*Insignificant associations*																
Prostate cancer	Mahmoud 2016	Diet	Adults	11,689/111,199	RR^a^	1.07	0.98, 1.64	17	3	13	0	1	Random	23.8	0.125	0.679
Prostate cancer	Mahmoud 2016	Diet	Adults	10,898/104,404	RR^d^	1.07	0.90, 1.28	12	3	9	0	0	Random	NA	NA	0.84
Gastric cancer	Li 2014	Diet	Adults	NA/4,128	RR^a^	0.91	0.64, 1.29	7	0	7	0	0	Random	77.6	92.2	NA
Esophageal cancer	Ma 2018	Diet	Adults	2,672/55,154	OR^a^	0.83	0.59, 1.16	11	2	9	0	0	Random	71	<0.001	No

*CI, confidence interval; NA, not available; OR, odds ratio; RCT, randomized controlled trial; RR, relative risk*.

a *Highest vs. lowest*.

b *<20mg/day vs. never*.

c *5 mg/day zinc increase.*

d *100mg/day zinc increase*.

### Mortality

On one hand, the higher dose of dietary zinc intake (40 mg/day) might not relate to all-cause mortality in adults (RR: 0.90, 95% CI: 0.63, 1.16) (27). On the other hand, low doses of zinc supplementations (10 mg/day) were not connected with decreased all-cause mortality in children (28). Concerning the survival and mortality of COVID-19, no significant findings favored the effect of zinc supplementation for them, including the survival to hospital discharge (Risk difference (RD): 0.01, 95% CI: −0.07, 0.08) and in-hospital mortality (RD: −0.03, 95% CI: −0.09, 0.03) (29).

### Cancer Outcomes

In adults, the highest vs. lowest dose of dietary zinc intakes were related to a reduction in the risk of suffering overall digestive cancer (30), colorectal cancer (30), and pancreatic cancer (31). However, compared with the lowest zinc intakes, reduced risk of prostate cancer (32), gastric cancer (30), and esophageal cancer (33) would not benefit from the highest zinc intakes. Furthermore, while dose-response calculation demonstrated that a daily increment of 100 mg zinc intakes was not linearly connected with the incidence of prostate cancer (RR: 1.07, 95% CI: 0.90, 1.286) (32), adults might get a 14% lower risk of colorectal cancer (RR: 0.86, 95% CI: 0.78, 0.96) (34) and 15% lower risk of esophageal cancer (RR: 0.86, 95% CI: 0.77, 0.96) (33) with an increment of 5 mg dietary zinc per day.

### Maternal and Associated Outcomes

Pregnant women who consumed a low dose of zinc supplementations (6–30 mg/day) might be related to a further decreased risk of childhood wheeze (RR: 0.57, 95% CI: 0.40, 0.81), but not childhood eczema (RR: 1.00, 95% CI: 0.69, 1.45) (35). Lower intakes of supplementary zinc were not associated with stillbirth, neonatal death, or mid-upper arm circumference (MUAC) of neonates (36). Similarly, higher doses of zinc supplementations in pregnancy (25-50 mg/day) might not attenuate low the risk of birthweight (17, 36), small for gestational age (37), pre-eclampsia or eclampsia (37), preterm birth (37), neonatal sepsis (36), or head circumference of neonates (36).

### Growth Outcomes

Zinc supplementations in children might be connected with a significant increase in height gain (15). This meta-analysis containing over 10,000 children in poverty-stricken countries from 19 RCTs manifested a gain of 0.43 (95% CI: 0.16, 0.70) cm in supplementary zinc group compared with no zinc intakes. At the same time, daily consumption of a low dose of zinc supplementations for children was also related to height (17), weight (17), weight gain (15), head circumference (15), weight-for-age z-scores (WAZ) (17), and weight-for-length z-scores (38). However, supplementary zinc intakes in low doses were not a strong indicator for the risk of stunting (RR: 1.00, 95% CI: 0.95, 1.06), underweight (RR: 1.08, 95% CI: 0.96, 1.21) and wasting (RR: 0.94, 95% CI: 0.82, 1.06) in children (15).

Compared with no zinc intakes, the effect from lower supplementary zinc intakes could consistently be applied to other physical development parameters, including head circumference change (15), height-for-age z-scores (HAZ) change (15), MUAC (16), MUAC change (15), WAZ change (15), HAZ (15, 16), weight for height z-scores (16) and psycho-motor development (39) as well as adulthood body fat percentage (40), body mass index (BMI) change (40), hip circumference (40), waist circumference change (40) and waist-to-hip ratio (40).

Apart from the above-mentioned, a daily dosage of <20 mg of supplementary zinc intakes would not benefit childhood mental development executive function (7), intelligence (7), and mental development index (16).

### Metabolic Outcomes

Supplementary zinc intakes would significantly level up serum zinc concentration in the general population (41) and decrease the incidence of childhood zinc deficiency by 63% (RR: 0.37, 95% CI: 0.22, 0.62) (16). For adults specifically, the zinc supplemented group though did help improve serum zinc concentration by 0.43 μmol/L, the negative value of the lower limit of the effect size manifested that there is a chance that zinc supplementation had no effect (37).

In terms of dietary intake in adults, the highest dose compared with the lowest dose might be associated with a 13% lower risk of type 2 diabetes mellitus (T2DM) (OR: 0.87, 95% CI: 0.78, 0.98) (42). Furthermore, daily supplementary zinc intake of more than 20 mg would relate to increasing total antioxidant capacity and glutathione, reducing malondialdehyde and serum inflammation factors including tumor necrosis factor-alpha (TNF-α), C-reactive protein (CRP) levels, but not interleukin-6 (IL-6) or nitric oxide (NO) (43, 44). In lipid metabolism among the general population, a high dosage of zinc supplementations would relate to a decrease of 10.92 mg/dl (95% CI: −18.56, −3.28), 6.87 mg/dl (95% CI: −11.16, −2.58) and 10.29 mg/dl (95% CI: −15.33, −6.52) for triglyceride, low density lipoprotein cholesterol and total cholesterol (TC) respectively, while an unobvious rise of high-density lipoprotein cholesterol (MD: 2.12, 95% CI: −0.74, 4.98) was also identified (45).

Besides, zinc supplementations were associated with a higher concentration of insulin-like growth factors-1 (IGF-1) (WMD: 8.62 ng/ml, 95% CI: 1.13, 16.11) in children and adults (46), while not for brain-derived neurotrophic factor levels (SMD: 0.30 μg/ml, 95% CI: −0.08, 0.67) (47) as well as leptin levels (WMD: 0.74 ng/ml, 95% CI: −1.39, 2.87) (48) in adults.

### Reproductive Outcomes

Supplementary zinc with a daily dosage of more than 20 mg was not connected to better sperm viability (SMD: −4.95, 95% CI: −9.87, −0.03) or increased sperm count (SMD: −4.95^*^10^6^/ml, 95% CI: −9.87, −0.03) (49). However, in comparison with no intakes, adequate zinc supplementations for adults improved sperm motility (MD: 7.03, 95% CI: 6.03, 8.03) (50), concentration (MD: 1.48^*^10^6^/ml, 95% CI: 0.69, 2.27) (50), morphology (SMD: −0.75%, 95% CI: −1.37, −0.14) (49) as well as volume (SMD: −0.99 ml, 95% CI: −1.60, −0.38) (49). Thus, the clinical pregnancy rate was found to drastically increase by 343% (OR: 4.43, 95% CI: 1.39, 14.14) (51).

### Respiratory Outcomes

With supplementary zinc intakes <20 mg per day, the incidence of childhood acute lower respiratory infections was lowered by 35% (RR: 0.65, 95% CI: 0.52, 0.82) (20). To be more specific, the incidence and prevalence of pneumonia were 13% (RR: 0.87, 95% CI: 0.81, 0.94) and 41% (RR: 0.59, 95% CI: 0.35, 0.99) lower in the zinc supplemented children, respectively (52). However, low dosages of zinc failed to neither significantly attenuate the risk of lower respiratory tract infections (RR: 0.78, 95% CI: 0.49, 1.24) (16) nor overall respiratory tract infection (RR: 0.91, 95% CI: 0.82, 1.01) (19) in children.

After receiving supplementary zinc over 20 mg each day, adults with acute viral respiratory tract infections (including COVID-19) would be 1.83 times more likely to recover before the placebo (HR: 1.83, 95% CI: 1.07, 3.13) as well as have a clinically significant reduction in mean duration (MD: −2.05, 95% CI: −3.50, −0.59) and day 3 symptom scores (MD: −1.20, 95% CI: −1.74, −0.66) (10). Yet average daily acute viral respiratory tract infection symptom scores in adults (MD: −0.15, 95% CI: −0.43, 0.13) (10) and symptoms of the common cold in the first week in the general population (OR: 0.52, 95% CI: 0.25, 1.20) (53) might not get improvement when they were supplemented with zinc.

### Neurologic Outcomes

Dietary zinc consumption of the highest dosage was related to a 34% lower risk of depression in adults (RR: 0.67, 95% CI: 0.58, 0.76) (54). At the same time, zinc supplemented depressed patients got a curative effect based on the fact that zinc could significantly lower down the depression symptom scores (WMD: −4.15 point, 95% CI: −6.56, −1.75) (55). In terms of another disease derived from neurologic dysfunction, the incidence of Parkinson's disease was not related to dietary zinc either from highest dosages vs. lowest dosages (RR: 0.89, 95% CI: 0.36, 2.18) or high dosages vs. no intake (RR: 0.69, 95% CI: 0.39, 1.23) (56).

### Digestive Outcomes

For children, low intakes of zinc supplements would decrease the incidence of diarrhea (RR: 0.89, 95% CI: 0.82, 0.97) (16) but not hyperbilirubinemia (OR: 1.14, 95% CI: 0.74, 1.76) (57).

### Skeletal Outcomes

There were significant associations between the zinc supplementations and alkaline phosphatase levels, osteocalcin levels, not parathyroid hormone levels, and bone alkaline phosphatase levels (58). It was the mineral density of femoral neck bone rather than lumbar bone that could benefit from zinc supplementations consumption among the general population (58). In addition, adults receiving dietary zinc might not significantly improve the incidence of overall bone health complications (MD: −0.33, 95% CI: −0.77, 0.11) (58).

### Other Outcomes

Supplementary zinc consumption lower than 20 mg per day might not improve adult tinnitus (RR: 2.53, 95% CI: 0.50, 12.70) (59). The same effect of zinc supplements worked for the incidence of malaria (60), anemia (16) and, otitis media (61) in children.

### Adverse Outcomes

Less than 20 mg zinc supplementations in children might be related to an obvious uprise of vomiting incidence (RR: 1.68, 95% CI: 1.61, 1.75) and vomiting prevalence (RR: 1.29, 95% CI: 1.14, 1.46) (60). Similarly, adults taking supplementary zinc for preventing or treating acute viral respiratory tract infections would also suffer a higher risk of taste aversion (RR: 2.11, 95%CI: 1.47, 3.04), mouth soreness (RR: 1.55, 95%CI: 1.05, 2.29), and gastrointestinal discomfort or nausea (RR: 1.46, 95% CI: 1.03, 2.06) (10).

### Heterogeneity of Included Studies

Approximately 27.91% of all included studies had a low degree of heterogeneity with I^2^ <25%; 34.88 and 33.56% of the meta-analyses had moderate and high heterogeneity, which containing I^2^ ranging from 25 to 75% and >75%, respectively. However, 4.65% of the studies did not report the heterogeneity and therefore could not be reanalyzed.

### Publication Bias of Included Studies

Twenty-one studies (48.84%) reported that there was no publication bias and 9 of them presented the exact Egger test value. 3 studies reported that there was statistically significant publication bias, including CRP levels (*p* = 0.002) (43), BMI change (*p* = 0.002) (40), height (*p* = 0.01) (17), weight (*p* = 0.03) (17) and WAZ (*p* = 0.04) (17). However, 19 studies did not report or mention the publication bias owing to the limited magnitude.

### AMSTAR2 and GRADE Evaluation of Included Studies

The results of AMSTAR2 were displayed in Supplementary Table 3. The vast majority (60.47%) of included articles were rated as “Critically Low” when only 34.88% and 4.65% of articles were rated as “Low” and “Moderate,” respectively. This was largely caused by the fact that most articles did not display the information of excluded studies, which was one of the critical domains. A detailed version of AMSTAR2 classification was in Supplementary Table 4. In terms of GRADE categorizations in Supplementary Table 3, 65.12% of the studies were “Very low,” 16.28% were “Low,” 6.98% were “Moderate” and 11.63% were “High.” The reason for the universally low evidence of strength was that on the one hand, the bias of studies was always unignorable and on the other hand, most studies failed to reach the width, breadth, and magnitude of the bonus items. The detailed information was displayed in Supplementary Table 5.

## Discussion

### Main Findings and Possible Explanations

In summary, 40 meta-analyses containing 93 unique outcomes for dietary and supplementary zinc consumption were identified in this umbrella review. Compared with the lowest dosage, dietary zinc intake in the highest dosage might reduce the incidence of digestive tract cancers, colorectal cancer, pancreatic cancer, T2DM, and depression in adults. 5 mg increment of zinc element might decrease the incidence of colorectal cancers and esophageal cancers. But higher zinc supplementation might not decrease all-cause mortality in adults or the in-hospital mortality of COVID-19. Supplementary zinc consumption <20 mg per day in adults was significantly linked with improvement of depression symptoms when intakes more than 20 mg per day might be related to better sperm quality, higher serum zinc concentration, increased pregnancy rate, and decreased concentration of inflammatory markers. For pregnant women, a low dosage of zinc supplementation in pregnancy would decrease the risk of further childhood wheeze. And zinc supplementation with a daily dose of <20 mg attenuated the incidence of acute lower respiratory infection, diarrhea, and pneumonia, the prevalence of pneumonia, increased zinc concentration, and decreased zinc deficiency as well as boosted growth in children. Furthermore, supplementary zinc intake might level up bone turnover markers, increase femoral bone mineral density, regulate blood lipids and increase serum IGF-1 in the general population.

Zinc concentration varies in organs, from up to 200 μg/g in the prostate, pancreas, and bone to down to 1 μg/g in the brain and plasma (62). Though plasma/serum zinc is known to contain only 0.1% of body zinc (63), it constitutes to be the most widely accepted biomarker of zinc as it was responsive to both zinc supplementation and depletion (64). In terms of dietary and supplementary zinc intake, zinc absorption in the human body is in reality influenced by multiple factors. Amount of zinc intake, the bioavailability of zinc compound (13), diseases including consistent diarrhea, genetic diseases and nutrient deficiencies (65), the existence of promoters and inhibitors (12), physiologic state, age stage, and inflammation would multi-dimensionally affect the zinc absorption. Even worse, zinc absorption fraction in humans and animals generally has an inverse association with the amount of zinc intake within a normal range (12, 13). Hence it is noteworthy that there is a disparity in the amount of zinc from intake and absorption. A meta-analysis containing five studies found that daily zinc supplements may improve maternal zinc concentrations although a lower limit just crossed the no-effect line (MD: 0.43 μmol/L, 95% CI: −0.04, 0.89) (37). In the general population, Furihata pointed out that overall zinc supplementation would elevate the serum zinc level by an average number of 9.08 μg/dL (95% CI: 5.46, 12.70) from baseline (41). Subgroup analysis also supported this impact. Another study conducted by Tam (16) pointed out that supplementary zinc intakes in children <20 mg daily would elevate the zinc concentration by 3.85 μmol/L (95% CI: 2.48, 5.23) and attenuate the risk of zinc deficiency by 63% (RR: 0.37, 95% CI: 0.22, 0.62). As such, it is generally believed that those with a higher intake are prone to have a proportionally higher net zinc absorption (12). Thus, dietary and supplementary zinc intake could be utilized as a noninvasive biomarker to evaluate the status of zinc and explore the impact on health outcomes.

However, zinc deficiency is prevalent around the world (~7.5–30%) (66), causing a substantial disease load in developing countries and low-income countries. Basically, it would be inappropriate to set a specific dose for adults, pregnant women, and children regardless of nutrition or disease status. However, zinc demand would be satisfied with an approximate recommended daily intake of 15 mg and the tolerable upper limit is 25 mg per daily in a healthy adult (67). A daily recommended intake of zinc is 20 mg per day for a pregnant woman (68). During infancy, daily zinc dosage ranges from 1 to 5 mg approximately (69). Even so, infants demanding high doses of zinc for growth and development, children bearing nutrients deficiency and gastrointestinal diseases and, pregnant women requiring high doses on reproduction (70) were at higher risk of zinc deficiency. But just as mentioned above, zinc supplementations might increase the concentration of serum zinc both in adults and children as well as improve zinc deficiency in children. Of note, mild zinc deficiency could be improved by proper dietary or supplementary zinc intake. When it comes to moderate or severe zinc deficiency, hosts were in extreme lack of zinc storage and might persist zinc depletion without sufficient zinc (13). Hence, it is strongly suggested to adhere to adequate zinc supplementation courses for them.

Serum zinc is thought to be the most important zinc pool for maintaining homeostasis, the majority of zinc yet combines to proteins as well as peptides and seems to be biologically inactive (71, 72). The mentioned proteins include albumin, α2-macroglobulin, and transferrin. These with different structural affinity to zinc ions can be regulated and altered by zinc concentration and consequently interact with cytokines and enzymes (62, 63, 73, 74). Throughout the cell, zinc metabolism can be divided into roughly three parts (3, 5, 12, 14): ZIP family transported zinc into the cytosol; ZnT family containing specific proteins exported zinc to extracellular space or organelles; metallothioneins (MTs) bind to 20% of intracellular total zinc and act as the buffer to reach zinc homeostasis. MTs bind to metals including copper, cadmium, zinc, and others and directly promote detoxification in a bid to counteract oxidative stresses and reduce cell apoptosis (74). As such, cellular zinc is considered as an indicator for apoptosis when the lower level might directly or indirectly relate to an increased number of cell apoptosis (3, 62). Lack of zinc might provoke a cascade of dysregulations of cell apoptosis, which feature in a series of diseases, such as autoimmune diseases, neurodegenerative diseases, and cancer. But the risk of Parkinson's disease from Cheng's observation could not be decreased through zinc supplementations (56). This study, however, reported limitations and bias including a small population and other confounding factors. A study in 2017 comprising 822 Parkinson's disease patients and 777 generally healthy individuals pointed out that serum zinc in Parkinson's disease patients was statistically lower than the level in the control group (SMD: −0.779 ug/g, 95% CI: −1.323, −0.234) (75). Parkinson's disease patients had relatively lower zinc levels both in cerebrospinal fluid and blood compared with healthy individuals was also reported in a recent meta-analysis (76). Similar conclusions mirrored those that were identified in the exploration of the zinc mechanism.

The immune function of humans could be influenced by zinc status. When zinc is diminished, humans will suffer depressed T and B lymphopoiesis as well as impaired maturation and antibody production (77, 78). Zinc deficiency would compromise the activities of natural killer cells, neutrophils, monocytes, macrophages, and T helper cells (5, 78–80). However, consistent daily intake of 20 mg zinc supplements in zinc-deficient children for 5 weeks would meet a percentage rise of CD4+ and CD8+ T cells (5). Older individuals with zinc deficiency following a seven-week duration of zinc consumption might increase the percentage of T helper lymphocytes (81). A large category of cytokines, including IL-4, IL-6, interferon-g (IFN-g), TNF-α from innate immunity (82), and IL-2, TNF, and IFN-γ from adaptive immunity (82, 83) were found to be inhibited in production in patients with zinc deficiency. Most of the cytokines mentioned were also in the participation of inflammation and oxidative stress (84, 85). A higher dose of zinc supplementations in adults could increase serum total antioxidant capacity (MD: 225.9 μmmol/L; 95% CI: 68.42, 383.5) (85) and glutathione (MD: 49.99 μmmol/L; 95% CI: 2.25, 97.73) (85), decrease malondialdehyde (ES: −0.42 nmol/ml, 95% CI: −0.83, −0.01) (43)and TNF-α (ES: −0.49 pg/mL, 95% CI: −0.84, −0.14) (43) and CRP (ES: −0.92 mg/L, 95% CI: −1.36, −0.48) (43). IL-6, also one of the biomarkers of the inflammation process, in Hosseini's study was found to marginally reduce 1.02 pg/mL compared with non-consumption even the upper limit crossed the no-effect line (ES: −1.02 pg/mL, 95% CI: −2.06, −0.02) (43). Interestingly, lower intake would decrease serum CRP concentration by 1.68 mg/L in a meta-analysis including 217 subjects and 200 controls (44). Another double-blinded study in 2015 found that after an 8-week intervention of 50 mg zinc daily, patients with polycystic ovary syndrome consumed only had a significant trend on reduced CRP levels (86). An important fact is that the improvement of oxidative stress comes from zinc-linked primary antioxidant enzymes (87). Zinc acts as a co-factor of superoxide dismutase, combining sulfhydryl groups and reducing the synthesis of intramolecular disulfide formation, and attacking oxidative damage (88). After binding to superoxide dismutase, zinc could also transform superoxide anion radicals into hydrogen peroxide (85). Other mechanisms to reduce oxidative stress and inflammation by zinc are as followed: stabilizing the cell membrane structure (43, 89, 90); suppressing the activity of NADPH (nicotinamide adenine dinucleotide phosphate) oxidase from producing superoxide anion radical (43, 91); down-regulating NF-κB which was activated by peroxisome proliferator-activated receptor-α to produce fewer cytokines (44); sustaining enough MTs to eliminate free radicals (72) and protecting the function of thiols (85, 91). Under these circumstances, it made sense that sperm concentration, morphology and volume and clinical pregnancy rate would see a rise when adults were heavily dosed (49–51).

And other beneficiaries who would benefit from the anti-oxidation and anti-inflammation effect of zinc included respiratory and digestive disorders containing pneumonia, gastroenteritis, and diarrhea (16, 20, 52, 53). Of note, the higher concentration of intracellular zinc could attenuate the multiplication of the RNA viruses including SARS-CoV-2, the pathogen of COVID-19 (92). The invasion and replication of SARS-CoV-2 required essential proteins included angiotensin-converting enzyme 2 in the cell membrane and RNA-dependent RNA polymerase and 3C-like proteinase in the nucleus (10, 93, 94). While Sirtuin 1 could linearly regulate the expression of angiotensin-converting enzyme 2, zinc was found to inhibit the expression of Sirtuin 1 and consequent angiotensin-converting enzyme 2 in the membrane, which could be the possible mechanism of reducing the invasion of COVID-19 (95). And intracellular zinc might inhibit the replication of COVID-19 through binding to the conserved binding sites of intranuclear enzymes (94, 96). Yet until now, no solid evidence-based data proved that zinc could further improve the outcome of patients with COVID-19 (10, 29). Further studies are needed to investigate the potential association between zinc therapy and COVID-19 infection.

Another critical problem to handle is diarrhea in children, especially in low-income countries. Not only zinc could boost the antioxidant response to cope with pathogens (97), but also maintain the integrity of the cell membrane (98) and block the potassium secretion through chlorine secretion (98). In this way, diarrhea in children can be to some certain extent inhibited by oral zinc in the intestines. Importance should be attached to zinc supplementation for the prevention and treatment of childhood diarrhea.

Concerning cancer, several mechanisms may contribute to the effect that zinc could to a great extent make the general population less cancer-prone. To start with, increased immunity and decreased oxidative stress from zinc make people less vulnerable to cancer as mentioned. Furthermore, the activity of many cancer-related enzymes (thymidine kinase, RNA and DNA polymerase, and so on) is zinc-dependent (5). Meanwhile, zinc is instrumental in interacting with zinc-binding domains, most notably zinc fingers (99). For instance, P53 suppressor protein and caspase-6 both partaking of zinc fingers, are critical in tumorigenesis. The former one is able to excise bases or nucleotides (100), and the latter cleaves the proenzyme form of caspase-3 and lamins and then ends up involving nuclear membrane dissolution (74). By inducing cell cycle arrest in the G2/M phase, zinc can also inhibit the proliferation of some kinds of cancer cells (101). More importantly, MT, especially ZIP is upregulating in cancer cells. It is considered critical in carcinogenesis, tumor prognosis, and metastasis (5, 62, 72, 74), thereafter elevating the zinc concentration inside and reducing the concentration outside. In clinical practice, the majority of cancer patients generally feature a decreased serum zinc compared with healthy ones (31, 99, 100). This was corroborated by a large-scale study among white Americans during 1970 and 1994 (102). The findings from Li in 2014 also agreed on these potential mechanisms (30). People would be less susceptible to the incidence of digestive tract cancers (RR: 0.82, 95% CI: 0.70, 0.96) and colorectal cancer (RR: 0.80, 95% CI: 0.69, 0.92) specifically when with the highest vs. lowest dietary intake. The risk of pancreatic cancer would be decreased with the highest dietary zinc intake (31). But Li also demonstrated that neither the risk of gastric and esophageal cancer would be reduced under the same circumstance. In a meta-analysis of over 50,000 participants, people would get an insignificantly protective effect from the highest zinc consumption (RR: 0.83, 95% CI: 0.59, 1.16) (33). Though statistically insignificant, subgroup and sensitivity analysis in both studies found that the degree of zinc deficiency and the food sources in different regions might explain the final effect. Interestingly, prostate cancers were shown to be an exception among cancers. Prostate cancer cells partake of lower zinc concentration in the cytoplasm in comparison with the controls. In this review, the fact that neither the highest zinc nor zinc increment was beneficial to the improvement of the risk of prostate cancer justified the mechanism (32).

Even though zinc supplementations could strengthen immunity multidimensionally, we should attach importance to the fact that supplementary zinc intakes could insignificantly decrease the risk of all-cause mortality in the general population (103). Simultaneously, subgroup analysis also displayed that breast cancer patients with referent dietary zinc intake would experience an unobvious 21% lower risk of overall survival (RR: 0.79, 95% CI: 0.56, 1.13) as well as a 21% lower risk of recurrence (RR: 0.79, 95% CI: 0.49, 1.28) (103).

Regarding growth, on one hand, zinc intakes in pregnancy are thought to relate to the better outcomes of children. Zinc deficiency dysregulated synthesis of nucleic acids and protein and then impaired cellular growth as described before. Also, decreased zinc level is related to increased chromosomal defects and lipid oxidation of cell membranes (104). When lung, heart, skin, urogenital system, and skeletal system suffer from these abnormalities, people in particular children and infants, would experience delayed or impaired growth. It is reported that pregnant women supplemented with zinc were liable to attenuate the risk of further childhood wheeze (RR: 0.57, 95% CI: 0.40, 0.81) but not childhood eczema (RR: 1.00, 95% CI: 0.69, 1.45) (35). But quite a few studies found an insignificant relationship between zinc supplementation and maternal outcomes. Ota et al. revealed that zinc supplementations either in low or high dosage were not significantly associated with a series of parameters including stillbirth, neonatal death, neonatal sepsis, high birthweight, MUAC, and head circumference of neonates (36). Similarly, low birthweight was not influenced by zinc (mean dose: 26.8mg/d) from a meta-analysis focused on infants (RR: 0.76, 95% CI: 0.52, 1.11) (17). In 2020, a meta-analysis established in low- and middle-income countries covering 439,649 women of varying gestational age pointed that a large dose of daily zinc supplementation could not attenuate the risk of suffering preterm birth, pre-eclampsia, or eclampsia (37). The reason why dietary and supplementary zinc is not proved to ameliorate the situation of maternal and associated outcomes were concluded as follows: first, most included studies focused on regions where zinc deficiency was prevalent while excluding other regions (37); second, in developing and low-income countries, other concurrent nutrient deficiencies were also present in most women (104); third, supplementation with a low dose of zinc was insufficient for better maternal outcomes when it was estimated that approximately 82% of the pregnant women around the world consumed zinc less the required dose (105, 106). On the other hand, zinc may positively boost children growth, mainly due to its direct impact on nucleic acid and protein synthesis (107), and hormonal mediators of growth (46); and its effects on appetite (108) and the risk of infection (106, 108) were also modifiers for growth outcomes. Specifically, in terms of hormonal mediators, normal bone metabolism and growth were contributed by growth hormone, IGF-1, and insulin-like growth factor-binding protein 3 (46, 109). In the general population, zinc supplementation would elevate the serum level of IGF-1 from the meta-analysis of Guo's (46). A very recent review showed that zinc supplemented human was more likely to meet a higher level of alkaline phosphatase (MD: 33.70 U/L, 95% CI: 22.79, 44.61), lower level of osteocalcin (MD:−4.14 ng/ml, 95% CI: −6.92, −1.36) as well as increased femoral neck bone mineral density (MD: 0.02 g/cm^2^, 95% CI: 0.01, 0.02) (58). But bone alkaline phosphatase level, bone alkaline phosphatase level, bone mineral density at the lumbar site, and overall bone health complications were not significantly improved by zinc supplementations. Gera evaluated the effect of zinc supplementation provided during childhood in a meta-analysis, but only to find that supplementations did improve the head circumference, weight gain, and height gain but not head circumference change, HAZ change, WAZ change, weight for height z-scores change, MUAC change as well as underweight, stunting and wasting (15). Meanwhile, a review in 2018 grossly approved the conclusion that zinc supplemented children were not improved in HAZ score, weight for height z-scores, stunting, underweight or wasting while getting a better outcome on height, weight, and WAZ score (17). The fact that children in developing countries were more zinc-deficient as mentioned above might justify the conclusion.

The central nervous system would also benefit from zinc intake (110). It is hypothesized that zinc could strengthen the activity of DNA/RNA polymerase in neurons as well as protect cell membranes from oxidative stress and inflammation. Other mechanisms include regulating neurotransmitters activities, increasing gamma-aminobutyric acid (111), brain-derived neurotrophic factor (112) through serotonergic, dopaminergic, and glutamatergic systems as well as mimicking the antidepressants' activities to normalize brain function (113, 114). Therefore, zinc might relate to the improvement of mood-related disorders and neurodegenerative diseases (115). According to Li's analysis from nine studies, the highest dietary zinc dosage could reduce the incidence of depression by 33% (95% CI: 0.58, 0.76) compared with the lowest zinc intake (54). The risk of depression was found lower with zinc supplementations (RR: 0.66, 95% CI: 0.50, 0.82), when the depressive symptom scores would be simultaneously lower than those in the control group only if given zinc monotherapy (WMD: −4.15 point, 95% CI: −6.56, −1.75) (55). However, due to the small magnitude of a meta-analysis, Jafari et al. found out that adults taking supplementary zinc for 12 weeks failed to experience elevated blood brain-derived neurotrophic factor concentration (ES: 0.30 ng/ml, 95% CI: −0.08, 0.67) (47). Apart from derivative diseases, we can also firmly believe that mental and motor development are strengthened by zinc supplementations. Yet from Tam's, Warthon-Medina's and Sajedi's studies, no statistically significant correlations were found between supplementary zinc intake (<20 mg/d) and mental development (MD: −0.15 point, 95% CI: −2.38, 2.09) (16), intelligence (SMD: 0.00 point, 95% CI: −0.12, 0.13) (7) and psycho-motor development (SMD: 0.30 point, 95% CI: −0.24, 0.83) (39), respectively.

However, besides the beneficial effect, zinc could have unwanted side effects. The incidence of vomiting can be leveled up by 29% due to zinc supplementation (60) though the effect would not last long. On the other hand, it has been demonstrated that fractional absorption and absorbed zinc are determined by current zinc intakes rather than the long-term status or past intake (13). This means a zinc-deficient person cannot massively up-regulate the efficacy of zinc intake and improve the status quo. Overdosed zinc intake thus would have a proportional adverse outcome. Furthermore, one study demonstrated that 11 men receiving 300 mg zinc per day for 6 weeks had weakened reactivity of lymphocytes and phagocytosis of polymorphonuclear leukocytes (1, 116). In children, excess zinc consumption can suppress monocyte function the IFN-γ production (5). Caution should thereafter be attached to the large intake and supplement of zinc dosages in the general population.

### Strengths and Limitations

This umbrella review had systematically integrated the current evidence about the associations between zinc intakes and different health outcomes for the first time. We pioneered utilizing the AMSTAR2 (22) and GRADE classification systems (25) to evaluate the strength and quality of the evidence of all meta-analyses. Some possible limitations should be noted nevertheless. To begin with, a relatively large number of the meta-analyses were “Critically Low” in AMSTAR2 classification as well as “Very low” in GRADE categorizations. This phenomenon was largely caused by that many studies did not display the list of excluded articles, report the publication bias in the critical domain AMSTAR2. Studies failed to explain the selection of study type and provide the funding sources of included studies also lent themselves to rank higher in the AMSTAR2. For GARDE categorization, serious imprecision derived from limited studies numbers and population numbers as well as considerate confidence intervals and I^2^. The fact that few studies met the upgrading items including a relatively large magnitude of effect and beneficial plausible confounding factors also contributed to low evidence. Due to the different rating domains, there is no statistical correlation between AMSTAR2 and GRADE. Second, according to our selection criteria, some of the studies might be missed because they were less recent and contained a smaller number of participants compared with included meta-analyses. Of note, some subgroup analyses of included studies may also be missed due to incomplete details required by the umbrella review. Third, a proportion of the eligible meta-analyses included a small number of studies and populations, which probably cause publication bias. Forth, the period of receiving zinc may confound the related conclusion due to lack of consensual definition. Finally, zinc intakes are as described as before would be influenced by a great number of factors, therefore, there might be a disparity between oral zinc and absorbed zinc. Even so, most of the studies include generally healthy participants, which would minimize the possible bias, to a large extent.

## Conclusion

According to our umbrella review, proper but not excess zinc intake would benefit the general population. Dietary zinc intake might reduce the risk of digestive tract cancers, depression, and T2DM in adults. Supplementary zinc intake in adults might improve depression, sperm quality, and concentration as well as pregnancy rate when in children reduce the risk of diarrhea, pneumonia, improve zinc deficiency and promote growth. Overall respiratory tract infections (including COVID-19) were improved due to anti-viral, anti-oxidative, and anti-inflammatory effects. Beneficial associations were also found in bone formation, blood lipids, and IGF-1metabolism in the general population. No evidence up till now favored that zinc could improve in-hospital mortality of COVID-19 and all-cause mortality. We recommended an additional 5-mg increment of zinc for human beings as suggested in the dose-response analysis about a lower risk of colorectal and esophageal cancers. High-quality and large-scale prospective studies are required to confirm the conclusions in the future.

## Author Contributions

JL, LL, and DC designed research. BC and ZC conducted research. RW and QD analyzed data. QW supervised the whole research, and JL, DC, and YH wrote the paper. LL had primary responsibility for final content. All authors contributed to and revised the submitted version of the paper.

## Funding

This work was supported by the National Natural Science Foundation of China (Grant Number 82000721) and Program from Department of Science and Technology of Sichuan Province (Grant Number 2020YJ0054).

## Conflict of Interest

The authors declare that the research was conducted in the absence of any commercial or financial relationships that could be construed as a potential conflict of interest.

## Publisher's Note

All claims expressed in this article are solely those of the authors and do not necessarily represent those of their affiliated organizations, or those of the publisher, the editors and the reviewers. Any product that may be evaluated in this article, or claim that may be made by its manufacturer, is not guaranteed or endorsed by the publisher.

## Abbreviations

AMSTAR: assessing the methodological quality of systematic reviews
BMI: body mass index
CI: confidence interval
CRP: C-reactive protein
ES: effect size
GRADE, Grading of Recommendations Assessment, Development: and Evaluation
HAZ: height-for-age z-scores
IFN: interferon
IGF-1: insulin-like growth factors-1
IL: interleukin
MD: mean difference
MT: metallothionein
MUAC: mid-upper arm circumference
NA: not available
NADPH: nicotinamide adenine dinucleotide phosphate
OR: odds ratio
RCT: randomized controlled trial
RD: risk difference
RR: relative risk
SMD: standardized mean difference
T2DM: type 2 diabetes mellitus
TNF-α: tumor necrosis factor-alpha
WAZ: weight for-age z-scores
WMD: weighted mean difference
ZIP: zinc-iron permease
ZnT: zinc transporters.
