# Supplementary material for: Zinc Intakes and Health Outcomes: An Umbrella Review
Source: Front Nutr. 2022 Feb 8;9:798078. doi: 10.3389/fnut.2022.798078 (PMC8861317; doi:10.3389/fnut.2022.798078)
Supplement: Supplementary file 1 [file Table_1.docx]

| Outcome | Author-Year | Type | Population | No. of cases/total | Metrics | Estimates | 95%CI | No. of studies | Cohort | Case control | Cross-sectional | RCT | Effects model | I^2^ | Q test p value | Egger test p value |
| --- | --- | --- | --- | --- | --- | --- | --- | --- | --- | --- | --- | --- | --- | --- | --- | --- |
| Mortality outcomes |  |  |  |  |  |  |  |  |  |  |  |  |  |  |  |  |
| *Insignificant associations* |  |  |  |  |  |  |  |  |  |  |  |  |  |  |  |  |
| All-cause mortality | Jayedi 2018 | Diet | Adults | 1,220/11,353 | RR^a^ | 0.90 | 0.63, 1.16 | 3 | 3 | 0 | 0 | 0 | Random | 48 | 0.14 | NA |
| All-cause mortality | Kanellopoulou 2021 | Supplement | Adults | NA/4,382 | RR^b^ | 0.90 | 0.69, 1.17 | 2 | 2 | 0 | 0 | 0 | Random | 0 | 0.511 | NA |
| All-cause mortality | Tam 2020 | Supplement | Children | NA/76,900 | RR^b^ | 0.24 | 0.04, 1.62 | 3 | 0 | 0 | 0 | 3 | Random | 100 | <0.002 | NA |
| Survival to hospital discharge of COVID-19 | Szarpak 2021 | Supplement | Adults | 273/433 | RD^e^ | 0.01 | -0.07, 0.08 | 2 | 1 | 0 | 0 | 1 | Fixed | 0 | 0.88 | NA |
| In‑hospital mortality of COVID-19 | Szarpak 2021 | Supplement | Adults | 104/541 | RD^e^ | -0.03 | -0.09, 0.03 | 3 | 1 | 0 | 0 | 2 | Fixed | 45 | 0.35 | NA |
| Cancer outcomes |  |  |  |  |  |  |  |  |  |  |  |  |  |  |  |  |
| *Significant associations* |  |  |  |  |  |  |  |  |  |  |  |  |  |  |  |  |
| Colorectal cancer | Qiao 2013 | Diet | Adults | 5,676/350,307 | RR^c^ | 0.86 | 0.78, 0.96 | 6 | 6 | 0 | 0 | 0 | Random | 33.5 | 0.16 | No |
| Esophageal cancer | Ma 2018 | Diet | Adults | 1,513/51,628 | OR^c^ | 0.85 | 0.77, 0.93 | 5 | 1 | 4 | 0 | 0 | Random | 54.2 | 0.068 | No |
| Digestive tract cancers | Li 2014 | Diet | Adults | 10,675/395,222 | RR^a^ | 0.82 | 0.70, 0.96 | 19 | 6 | 13 | 0 | 0 | Random | 75.7 | <0.001 | No |
| Colorectal cancer | Li 2014 | Diet | Adults | NA/35,2319 | RR^a^ | 0.80 | 0.69, 0.92 | 6 | 5 | 1 | 0 | 0 | Random | 60.5 | 0.027 | NA |
| Pancreatic cancer | Li 2017 | Diet | Adults | 1,659/106,359 | RR^a^ | 0.798 | 0.621, 0.984 | 7 | 2 | 5 | 0 | 0 | Random | 58.2 | 0.026 | 0.997 |
| *Insignificant associations* |  |  |  |  |  |  |  |  |  |  |  |  |  |  |  |  |
| Prostate cancer | Mahmoud 2016 | Diet | Adults | 11,689/111,199 | RR^a^ | 1.07 | 0.98, 1.64 | 17 | 3 | 13 | 0 | 1 | Random | 23.8 | 0.125 | 0.679 |
| Prostate cancer | Mahmoud 2016 | Diet | Adults | 10,898/104,404 | RR^d^ | 1.07 | 0.90, 1.28 | 12 | 3 | 9 | 0 | 0 | Random | NA | NA | 0.84 |
| Gastric cancer | Li 2014 | Diet | Adults | NA/4,128 | RR^a^ | 0.91 | 0.64, 1.29 | 7 | 0 | 7 | 0 | 0 | Random | 77.6 | 92.2 | NA |
| Esophageal cancer | Ma 2018 | Diet | Adults | 2,672/55,154 | OR^a^ | 0.83 | 0.59, 1.16 | 11 | 2 | 9 | 0 | 0 | Random | 71 | <0.001 | No |
| Maternal and associated outcomes |  |  |  |  |  |  |  |  |  |  |  |  |  |  |  |  |
| *Significant associations* |  |  |  |  |  |  |  |  |  |  |  |  |  |  |  |  |
| Childhood wheeze | Beckhaus 2015 | Supplement | Adults | NA/3,315 | RR^b^ | 0.57 | 0.40, 0.81 | 3 | 3 | 0 | 0 | 0 | Fixed | 0 | 0.6 | NA |
| *Insignificant associations* |  |  |  |  |  |  |  |  |  |  |  |  |  |  |  |  |
| Stillbirth or neonatal death | Ota 2015 | Supplement | Adults | 214/5,100 | RR^b^ | 1.12 | 0.86, 1.46 | 8 | 0 | 0 | 0 | 8 | Fixed | 0 | 0.73 | NA |
| Small for gestational age | Oh 2020 | Supplement | Adults | 998/2,174 | RR^e^ | 1.05 | 0.97, 1.13 | 3 | 0 | 0 | 0 | 3 | Random | 0 | 0.37 | NA |
| Pre-eclampsia/eclampsia | Oh 2020 | Supplement | Adults | 36/1,226 | RR^e^ | 1.01 | 0.53, 1.93 | 3 | 0 | 0 | 0 | 3 | Random | 0 | 0.72 | NA |
| Childhood eczema | Beckhaus 2015 | Supplement | Adults | NA/7,741 | RR^b^ | 1.00 | 0.69, 1.45 | 5 | 5 | 0 | 0 | 0 | Fixed | 63 | <0.001 | NA |
| High birthweight | Ota 2015 | Supplement | Adults | 406/2,837 | RR^e^ | 1.00 | 0.84, 1.18 | 5 | 0 | 0 | 0 | 5 | Fixed | 28 | 0.23 | NA |
| Preterm birth | Oh 2020 | Supplement | Adults | 613/5,017 | RR^e^ | 0.97 | 0.80, 1.17 | 11 | 0 | 0 | 0 | 11 | Random | 22 | 0.23 | NA |
| Low birthweight | Liu 2018 | Supplement | Adults | NA/8,467 | RR^e^ | 0.76 | 0.52, 1.11 | 13 | 0 | 0 | 0 | 13 | Random | 91.1 | <0.001 | NA |
| MUAC of neonates | Ota 2015 | Supplement | Adults | 933/911^f^ | MD^b^ | 0.74 | -0.17, 1.65 | 3 | 0 | 0 | 0 | 3 | Fixed | 27 | 0.25 | NA |
| Neonatal sepsis | Ota 2015 | Supplement | Adults | 7/736 | RR^e^ | 0.17 | 0.03, 1.01 | 2 | 0 | 0 | 0 | 2 | Fixed | 0 | 0.79 | NA |
| Birthweight of neonates | Liu 2018 | Supplement | Adults | NA/13,167 | MD^e^ | 0.08 | -0.05, 0.22 | 22 | 0 | 0 | 0 | 22 | Random | 98 | <0.001 | NA |
| Head circumference of neonates | Ota 2015 | Supplement | Adults | 2,014/1,977^f^ | MD^e^ | -0.03 | -0.17, 0.11 | 7 | 0 | 0 | 0 | 7 | Random | 45 | 0.09 | NA |
| Growth outcomes |  |  |  |  |  |  |  |  |  |  |  |  |  |  |  |  |
| *Significant associations* |  |  |  |  |  |  |  |  |  |  |  |  |  |  |  |  |
| Height gain | Gera 2019 | Supplement | Children | 5,252/5,531^f^ | MD^b^ | 0.43 | 0.16, 0.70 | 19 | 0 | 0 | 0 | 19 | Random | 93 | <0.001 | No |
| Head circumference | Gera 2019 | Supplement | Children | 2,110/856^f^ | MD^b^ | 0.39 | 0.03, 0.75 | 6 | 0 | 0 | 0 | 6 | Random | 67 | <0.001 | No |
| Height | Liu 2018 | Supplement | Children | NA/14,131 | WMD^b^ | 0.23 | 0.09, 0.38 | 40 | 0 | 0 | 0 | 40 | Random | 66.9 | <0.001 | 0.01 |
| Weight-for-length z-scores | Lassi 2020 | Supplement | Children | 478/477^f^ | SMD^b^ | 0.15 | 0.02, 0.28 | 3 | 0 | 0 | 0 | 3 | Fixed | 0 | 0.81 | NA |
| Weight | Liu 2018 | Supplement | Children | NA/13,841 | WMD^b^ | 0.14 | 0.07, 0.21 | 39 | 0 | 0 | 0 | 39 | Random | 84.7 | <0.001 | 0.03 |
| Weight gain | Gera 2019 | Supplement | Children | 4,936/5,207^f^ | MD^b^ | 0.11 | 0.05, 0.17 | 23 | 0 | 0 | 0 | 23 | Random | 80 | <0.001 | No |
| WAZ | Liu 2018 | Supplement | Children | NA/14,329 | WMD^b^ | 0.04 | 0.001, 0.087 | 36 | 0 | 0 | 0 | 36 | Random | 67.2 | <0.001 | 0.04 |
| *Insignificant associations* |  |  |  |  |  |  |  |  |  |  |  |  |  |  |  |  |
| Underweight | Gera 2019 | Supplement | Children | 1,389/8,677 | RR^b^ | 1.08 | 0.96, 1.21 | 7 | 0 | 0 | 0 | 7 | Random | 73 | <0.001 | No |
| Stunting | Gera 2019 | Supplement | Children | 3,759/11,838 | RR^b^ | 1.00 | 0.95, 1.06 | 10 | 0 | 0 | 0 | 10 | Random | 11 | No | No |
| Wasting | Gera 2019 | Supplement | Children | 2,742/8,988 | RR^b^ | 0.94 | 0.82, 1.06 | 7 | 0 | 0 | 0 | 7 | Random | 13 | No | No |
| Psycho-motor development index | Sajedi 2020 | Supplement | Children | 497/480^f^ | SMD^b^ | 0.30 | -0.24, 0.83 | 6 | 0 | 0 | 0 | 6 | Random | 94 | <0.001 | No |
| Head circumference change | Gera 2019 | Supplement | Children | 254/243^f^ | MD^b^ | 0.26 | -0.18, 0.71 | 4 | 0 | 0 | 0 | 4 | Random | 79 | 0.003 | No |
| HAZ change | Gera 2019 | Supplement | Children | 4,271/4,581^f^ | MD^b^ | 0.11 | -0.00, 0.21 | 13 | 0 | 0 | 0 | 13 | Random | 94 | <0.001 | No |
| Executive function | Warthon-Medina 2015 | Supplement | Children | 635/766^f^ | SMD^b^ | 0.08 | -0.06, 0.22 | 4 | 0 | 0 | 4 | 0 | Random | 38 | 0.16 | NA |
| MUAC | Tam 2020 | Supplement | Children | 1,755/1,743^f^ | MD^b^ | 0.06 | -0.02, 0.15 | 10 | 0 | 0 | 0 | 10 | Random | 27 | 0.2 | NA |
| Hip circumference | Abdollahi 2020 | Supplement | Adults | NA/286 | WMD^e^ | 0.05 | -0.61, 0.71 | 4 | 0 | 0 | 0 | 4 | Random | 0 | 0.81 | NA |
| Waist-to-hip ratio | Mayo-Wilson 2014 | Supplement | Children | NA/7,901 | SMD^b^ | 0.05 | 0.01, 0.1 | 24 | 0 | 0 | 0 | 24 | Fixed | 20 | 0.17 | NA |
| WAZ change | Gera 2019 | Supplement | Children | 4,207/4,581^f^ | MD^b^ | 0.03 | -0.01, 0.08 | 13 | 0 | 0 | 0 | 13 | Random | 66 | <0.001 | No |
| Weight for height z-scores | Tam 2020 | Supplement | Children | 4,456/4,884^f^ | MD^b^ | 0.03 | -0.01, 0.08 | 18 | 0 | 0 | 0 | 18 | Random | 31 | 0.1 | NA |
| MUAC change | Gera 2019 | Supplement | Children | 2,729/1,507^f^ | MD^b^ | 0.01 | -0.03, 0.04 | 7 | 0 | 0 | 0 | 7 | Random | 18 | 0.29 | No |
| Weight for height z-scores change | Gera 2019 | Supplement | Children | 4,200/4,506^f^ | MD^b^ | 0.01 | -0.03, 0.04 | 12 | 0 | 0 | 0 | 12 | Random | 0 | 0.54 | No |
| BMI change | Abdollahi 2020 | Supplement | Adults | NA/1,230 | WMD^e^ | 0.00 | -0.17, 0.18 | 21 | 0 | 0 | 0 | 21 | Random | 56.9 | <0.001 | 0.002 |
| Intelligence | Warthon-Medina 2015 | Supplement | Children | 755/811^f^ | SMD^b^ | 0.00 | -0.12, 0.13 | 5 | 0 | 0 | 5 | 0 | Random | 33 | 0.19 | NA |
| HAZ | Tam 2020 | Supplement | Children | 4,613/5,048^f^ | MD^b^ | 0.00 | -0.03, 0.02 | 20 | 0 | 0 | 0 | 20 | Random | 0 | 0.64 | NA |
| Waist-to-hip ratio | Abdollahi 2020 | Supplement | Adults | NA/354 | WMD^e^ | 0.00 | -0.02, 0.01 | 5 | 0 | 0 | 0 | 5 | Random | 0 | 0.9 | NA |
| Waist circumference change | Abdollahi 2020 | Supplement | Adults | NA/456 | WMD^e^ | -0.09 | -0.66, 0.48 | 7 | 0 | 0 | 0 | 7 | Random | 0 | 0.97 | NA |
| Mental development index | Tam 2020 | Supplement | Children | NA/4,262^f^ | MD^b^ | -0.15 | -2.38, 2.09 | 4 | 0 | 0 | 0 | 4 | Random | 71 | 0.02 | NA |
| Body fat percentage | Abdollahi 2020 | Supplement | Adults | NA/335 | WMD^e^ | -0.30 | -1.48, 0.89 | 5 | 0 | 0 | 0 | 5 | Random | 0 | 0.57 | NA |

**Table S1. Associations between zinc intakes and maternal and growth outcomes.**

BMI, body mass index; CI, confidence interval; HAZ, height-for-age z-scores; MD, mean difference; MUAC, mid-upper arm circumference; NA, not available; OR, odds ratio; RCT, randomized controlled trial; RD, risk difference; RR, relative risk; SMD, standardize mean difference; WAZ, weight for-age z-scores; WMD, weighted mean difference.

^a^ Highest versus lowest

^b^ <20mg/day versus never

^c^ 5 mg/day zinc increase

^d^ 100mg/day zinc increase

^e^ >20mg/day versus never

^f^ Cases/control
