# Supplementary material for: Zinc Intakes and Health Outcomes: An Umbrella Review
Source: Front Nutr. 2022 Feb 8;9:798078. doi: 10.3389/fnut.2022.798078 (PMC8861317; doi:10.3389/fnut.2022.798078)
Supplement: Supplementary file 2 [file Table_2.docx]

| Outcome | Author-Year | Type | Population | No of cases/total | Metrics | Estimates | 95%CI | No of studies | Cohort | Case control | Cross-sectional | RCT | Effects model | I^2^ | Q test p value | Egger test p value |
| --- | --- | --- | --- | --- | --- | --- | --- | --- | --- | --- | --- | --- | --- | --- | --- | --- |
| Metabolic outcomes |  |  |  |  |  |  |  |  |  |  |  |  |  |  |  |  |
| *Significant associations* |  |  |  |  |  |  |  |  |  |  |  |  |  |  |  |  |
| Total antioxidant capacity | Mousavi 2020 | Supplement | Adults | 149/149^f^ | WMD^e^ | 226 | 68.42, 383.50 | 4 | 0 | 0 | 0 | 4 | Random | 92.3 | <0.001 | NA |
| Glutathione | Mousavi 2020 | Supplement | Adults | 126/126^f^ | WMD^e^ | 49.99 | 2.25, 97.73 | 3 | 0 | 0 | 0 | 3 | Random | 54.5 | <0.001 | NA |
| Change of zinc concentrations | Furihata 2020 | Supplement | Adults and children | 96/87^f^ | MD^e^ | 9.08 | 5.46, 12.70 | 3 | 0 | 0 | 0 | 3 | Fixed | 0.61 | 0.46 | NA |
| IGF-1 levels | Guo 2020 | Supplement | Adults and children | 247/218^f^ | WMD^b^ | 8.62 | 1.13, 16.11 | 10 | 0 | 0 | 0 | 10 | Random | 97.3 | <0.001 | 0.531 |
| Zinc concentrations | Tam 2020 | Supplement | Children | NA/62,146 | MD^b^ | 3.85 | 2.48, 5.23 | 19 | 0 | 0 | 0 | 19 | Random | 99 | <0.001 | NA |
| Type 2 Diabetes Mellitus | Fernandez-Cao 2019 | Diet | Adults | 11,511/146,117 | OR^a^ | 0.87 | 0.78, 0.98 | 7 | 6 | 0 | 1 | 0 | Random | 64.5 | 0.003 | 0.429 |
| Zinc concentrations | Oh 2020 | Supplement | Adults | NA/1,202 | MD^e^ | 0.43 | -0.04, 0.89 | 5 | 0 | 0 | 0 | 5 | Random | 79 | <0.001 | NA |
| Zinc deficiency | Tam 2020 | Supplement | Children | 953/3,040^f^ | RR^b^ | 0.37 | 0.22, 0.62 | 11 | 0 | 0 | 0 | 11 | Random | 93 | <0.001 | NA |
| Malondialdehyde | Hosseini 2021 | Supplement | Adults | NA/228 | ES^e^ | -0.42 | -0.83, -0.01 | 5 | 0 | 0 | 0 | 5 | Random | 76.1 | <0.001 | 0.32 |
| TNF-a | Hosseini 2021 | Supplement | Adults | NA/215 | ES^e^ | -0.49 | -0.84, -0.14 | 5 | 0 | 0 | 0 | 5 | Random | 34.6 | 0.191 | 0.68 |
| CRP levels | Hosseini 2021 | Supplement | Adults | NA/966 | ES^e^ | -0.92 | -1.36, -0.48 | 15 | 0 | 0 | 0 | 15 | Random | 90.2 | <0.001 | 0.002 |
| CRP levels | Mousavi 2018 | Supplement | Adults | 217/200^f^ | WMD^b^ | -1.68 | -2.40, -0.90 | 8 | 0 | 0 | 0 | 8 | Random | 82.2 | <0.001 | 0.672 |
| Low-density lipoprotein cholesterol | Ranasinghe 2015 | Supplement | Adults and children | 636/633^f^ | MD^e^ | -6.87 | -11.16, -2.58 | 17 | 0 | 0 | 0 | 17 | Random | 31 | 0.08 | NA |
| Total cholesterol | Ranasinghe 2015 | Supplement | Adults and children | 765/763^f^ | MD^e^ | -10.29 | -15.33, -6.52 | 24 | 0 | 0 | 0 | 24 | Random | 83 | <0.001 | NA |
| Triglyceride | Ranasinghe 2015 | Supplement | Adults and children | 666/651^f^ | MD^e^ | -10.92 | -18.56, -3.28 | 19 | 0 | 0 | 0 | 19 | Random | 69 | <0.001 | NA |
| *Insignificant associations* |  |  |  |  |  |  |  |  |  |  |  |  |  |  |  |  |
| High density lipoprotein cholesterol | Ranasinghe 2015 | Supplement | Adults and children | 755/753^f^ | MD^e^ | 2.12 | -0.74, 4.98 | 21 | 0 | 0 | 0 | 21 | Random | 83 | <0.001 | NA |
| Serum leptin levels | Khorshidi 2019 | Supplement | Adults | 121/123^f^ | WMD^e^ | 0.74 | -1.39, 2.87 | 7 | 0 | 0 | 0 | 7 | Random | 81.7 | <0.001 | 0.53 |
| Brain-derived neurotrophic factor levels | Jafari 2021 | Supplement | Adults and children | NA/238 | SMD^e^ | 0.30 | -0.08, 0.67 | 5 | 0 | 0 | 0 | 5 | Random | 50 | 0.091 | No |
| IL-6 level | Hosseini 2021 | Supplement | Adults | NA/424 | ES^e^ | -1.02 | -2.06, 0.02 | 5 | 0 | 0 | 0 | 8 | Random | 92.3 | <0.001 | NA |
| Nitric oxide level | Mousavi 2020 | Supplement | Adults | 113/109^f^ | WMD^e^ | -1.66 | -5.89, 2.58 | 4 | 0 | 0 | 0 | 4 | Random | 68.1 | 0.02 | NA |
| Reproductive outcomes |  |  |  |  |  |  |  |  |  |  |  |  |  |  |  |  |
| *Significant associations* |  |  |  |  |  |  |  |  |  |  |  |  |  |  |  |  |
| Sperm motility | Salas-Huetos 2018 | Supplement | Adults | 119/116^f^ | MD^e^ | 7.03 | 6.03, 8.03 | 3 | 0 | 0 | 0 | 3 | Fixed | 1 | <0.001 | NA |
| Clinical pregnancy rate | Smits 2019 | Supplement | Adults | 90/63^f^ | OR^e^ | 4.43 | 1.39, 14.14 | 2 | 0 | 0 | 0 | 2 | Fixed | 0 | 0.94 | NA |
| Sperm concentration | Salas-Huetos 2018 | Supplement | Adults | 119/116^f^ | MD^e^ | 1.48 | 0.69, 2.27 | 3 | 0 | 0 | 0 | 3 | Fixed | 0 | <0.001 | NA |
| Sperm morphology | Zhao 2016 | Supplement | Adults | 138/138^f^ | SMD^e^ | -0.75 | -1.37, -0.14 | 4 | 0 | 4 | 0 | 0 | Random | 82 | <0.001 | NA |
| Sperm volume | Zhao 2016 | Supplement | Adults | 506/506^f^ | SMD^e^ | -0.99 | -1.60, -0.38 | 5 | 0 | 5 | 0 | 0 | Random | 91 | <0.000 | NA |
| *Insignificant associations* |  |  |  |  |  |  |  |  |  |  |  |  |  |  |  |  |
| Sperm count | Zhao 2016 | Supplement | Adults | 444/444^f^ | SMD^e^ | -4.95 | -9.87, -0.03 | 3 | 0 | 3 | 0 | 0 | Random | 100 | <0.001 | NA |
| Sperm viability | Zhao 2016 | Supplement | Adults | 444/444^f^ | SMD^e^ | -4.95 | -9.87, -0.03 | 3 | 0 | 3 | 0 | 0 | Random | 100 | <0.001 | NA |
| Respiratory outcomes |  |  |  |  |  |  |  |  |  |  |  |  |  |  |  |  |
| *Significant associations* |  |  |  |  |  |  |  |  |  |  |  |  |  |  |  |  |
| Remaining acute viral respiratory tract infection symptoms over 7 days | Hunter 2021 | Supplement | Adults | 527/496 ^f^ | HR^e^ | 1.83 | 1.07, 3.13 | 7 | 0 | 0 | 0 | 7 | Random | 82 | 0.03 | No |
| Pneumonia | Lassi 2010 | Supplement | Children | 3,922/3,928^f^ | RR^b^ | 0.87 | 0.81, 0.94 | 6 | 0 | 0 | 0 | 6 | Fixed | 28 | 0.2 | No |
| Acute lower respiratory infection | Roth 2010 | Supplement | Children | 759/4,712 | RR^b^ | 0.65 | 0.52, 0.82 | 3 | 0 | 0 | 0 | 3 | Random | 26.6 | 0.256 | No |
| Pneumonia prevalence | Lassi 2010 | Supplement | Children | 1,633/1,633^f^ | RR^b^ | 0.59 | 0.35, 0.99 | 1 | 0 | 0 | 0 | 1 | Fixed | NA | NA | NA |
| Acute viral respiratory tract infection symptom in day 3 | Hunter 2021 | Supplement | Adults | 200/192 ^f^ | MD^e^ | -1.20 | -1.74, -0.66 | 5 | 0 | 0 | 0 | 5 | Random | 0 | <0.001 | No |
| Mean duration of acute viral respiratory tract infection symptom | Hunter 2021 | Supplement | Adults | 607/573 ^f^ | MD^e^ | -2.05 | -3.50, -0.59 | 12 | 0 | 0 | 0 | 12 | Random | 97 | 0.006 | 0.54 |
| *Insignificant associations* |  |  |  |  |  |  |  |  |  |  |  |  |  |  |  |  |
| Respiratory tract infection | Vlieg-Boerstra 2021 | Supplement | Children | 51,290/51,344 ^f^ | RR^b^ | 0.91 | 0.82, 1.01 | 18 | 0 | 0 | 0 | 18 | Random | 83.7 | <0.001 | NA |
| Lower respiratory tract infection | Tam 2020 | Supplement | Children | NA/47,764 | RR^b^ | 0.78 | 0.49, 1.24 | 6 | 0 | 0 | 0 | 6 | Random | 98 | <0.001 | NA |
| Common cold symptom in 1st week | Jackson 2000 | Supplement | Adults and children | NA/890 | OR^b^ | 0.52 | 0.25, 1.20 | 8 | 0 | 0 | 0 | 8 | Random | NA | NA | 0.09 |
| Average acute viral respiratory tract infection symptom | Hunter 2021 | Supplement | Adults | 97/98 ^f^ | MD^e^ | -0.15 | -0.43, 0.13 | 3 | 0 | 0 | 0 | 3 | Random | 0 | 0.31 | No |
| Neurologic outcomes |  |  |  |  |  |  |  |  |  |  |  |  |  |  |  |  |
| *Significant associations* |  |  |  |  |  |  |  |  |  |  |  |  |  |  |  |  |
| Depression | Li 2017 | Diet | Adults | 3,768/22,158 | RR^a^ | 0.67 | 0.58, 0.76 | 9 | 3 | 1 | 5 | 0 | Random | 0 | 0.509 | 0.773 |
| Depression symptom scores | Yosaee 2020 | Supplement | Adults | 159/160 | WMD^b^ | -4.15 | -6.56, -1.75 | 7 | 0 | 0 | 0 | 7 | Random | 80.1 | <0.001 | NA |
| *Insignificant associations* |  |  |  |  |  |  |  |  |  |  |  |  |  |  |  |  |
| Parkinson's disease | Cheng 2015 | Diet | Adults | 499/1,255 | RR^a^ | 0.89 | 0.36, 2.18 | 2 | 0 | 2 | 0 | 0 | Random | 92.2 | <0.001 | NA |
| Parkinson's disease | Cheng 2015 | Diet | Adults | 499/1,255 | RR^e^ | 0.69 | 0.39, 1.23 | 2 | 0 | 2 | 0 | 0 | Random | 48.4 | 0.164 | NA |
| Digestive outcomes |  |  |  |  |  |  |  |  |  |  |  |  |  |  |  |  |
| *Significant associations* |  |  |  |  |  |  |  |  |  |  |  |  |  |  |  |  |
| Diarrhea | Tam 2020 | Supplement | Children | NA/53,322 | RR^b^ | 0.89 | 0.82, 0.97 | 11 | 0 | 0 | 0 | 11 | Random | 86 | <0.001 | NA |
| *Insignificant associations* |  |  |  |  |  |  |  |  |  |  |  |  |  |  |  |  |
| Hyperbilirubinemia | Yang 2018 | Supplement | Children | 110/477 | OR^b^ | 1.14 | 0.74, 1.76 | 3 | 0 | 0 | 0 | 3 | Fixed | 0 | 0.55 | No |
| Skeletal outcomes |  |  |  |  |  |  |  |  |  |  |  |  |  |  |  |  |
| *Significant associations* |  |  |  |  |  |  |  |  |  |  |  |  |  |  |  |  |
| Alkaline phosphatase level | Ceylan 2021 | Supplement | Adults and children | 40/40^f^ | MD^e^ | 33.70 | 22.79, 44.61 | 2 | 0 | 2 | 0 | 0 | Fixed | 0 | 0.61 | No |
| Femoral neck bone mineral density | Ceylan 2021 | Supplement | Adults and children | 234/249^f^ | MD^b^ | 0.02 | 0.01, 0.02 | 3 | 0 | 3 | 0 | 0 | Fixed | 85 | <0.001 | No |
| Osteocalcin levels | Ceylan 2021 | Supplement | Adults and children | 168/176^f^ | MD^b^ | -4.14 | -6.92, -1.36 | 4 | 0 | 4 | 0 | 0 | Random | 94 | <0.001 | No |
| *Insignificant associations* |  |  |  |  |  |  |  |  |  |  |  |  |  |  |  |  |
| Parathyroid hormone level | Ceylan 2021 | Supplement | Adults and children | 230/244^f^ | MD^b^ | 3.55 | -18.88, 25.98 | 4 | 0 | 4 | 0 | 0 | Random | 100 | <0.001 | No |
| Bone alkaline phosphatase level | Ceylan 2021 | Supplement | Adults and children | 56/70^f^ | MD^b^ | 0.84 | -3.96, 5.64 | 2 | 0 | 2 | 0 | 0 | Random | 77 | 0.04 | No |
| Lumbar bone mineral density | Ceylan 2021 | Supplement | Adults and children | 262/280^f^ | MD^b^ | -0.01 | -0.01, 0.00 | 4 | 0 | 4 | 0 | 0 | Fixed | 40 | 0.13 | No |
| Overall bone health complications | Ceylan 2021 | Diet | Adults and children | 1,606/9,389 | MD^b^ | -0.33 | -0.77, 0.11 | 12 | 1 | 10 | 1 | 0 | Random | 96 | <0.001 | No |
| Other outcomes |  |  |  |  |  |  |  |  |  |  |  |  |  |  |  |  |
| *Insignificant associations* |  |  |  |  |  |  |  |  |  |  |  |  |  |  |  |  |
| Tinnitus improvement | Person 2016 | Supplement | Adults | 7/187 | RR^b^ | 2.53 | 0.50, 12.70 | 1 | 0 | 0 | 0 | 1 | Fixed | NA | NA | NA |
| Malaria | Mayo-Wilson 2014 | Supplement | Children | NA/2,407 | RR^b^ | 1.05 | 0.95, 1.15 | 4 | 0 | 0 | 0 | 4 | Fixed | 0 | 0.84 | NA |
| At least one otitis media | Gulani 2012 | Supplement | Children | 224/3,191 | RR^b^ | 1.05 | 0.82, 1.36 | 2 | 0 | 0 | 0 | 2 | Fixed | 18 | 0.27 | No |
| Anemia | Tam 2020 | Supplement | Children | 1,623/3,147 | RR^b^ | 1.01 | 0.96, 1.06 | 10 | 0 | 0 | 0 | 10 | Random | 0 | 0.61 | NA |

**Table S2. Associations between zinc intakes and metabolic, reproductive, respiratory, neurologic, digestive, skeletal** **and other outcomes.**

CI, confidence interval; CRP, C-reactive protein; ES, effect size; IGF-1, insulin-like growth factors -1; IL, interleukin; MD, mean difference; NA, not available; OR, odds ratio; RCT, randomized controlled trial; RR, relative risk; SMD, standardize mean difference; TNF-a, tumor necrosis factor-alpha; WMD, weighted mean difference.

^a^ Highest versus lowest

^b^ <20mg/day versus never

^c^ 5 mg/day zinc increase

^d^ 100mg/day zinc increase

^e^ >20mg/day versus never

^f^ Cases/control
