# Supplementary material for: Zinc Intakes and Health Outcomes: An Umbrella Review
Source: Front Nutr. 2022 Feb 8;9:798078. doi: 10.3389/fnut.2022.798078 (PMC8861317; doi:10.3389/fnut.2022.798078)
Supplement: Supplementary file 3 [file Table_3.docx]

| Outcome | Assessed with | Author-Year | Type | AMSTAR2 | GRADE |
| --- | --- | --- | --- | --- | --- |
| Mortality outcomes |  |  |  |  |  |
| All-cause mortality | Highest versus lowest | Jayedi 2018 | Diet | Critically Low | Very low |
| All-cause mortality | <20mg/day versus never | Kanellopoulou 2021 | Supplement | Critically Low | Very low |
| All-cause mortality | <20mg/day versus never | Tam 2020 | Supplement | Low | Low |
| Survival to hospital discharge of COVID-19 | >20mg/day versus never | Szarpak 2021 | Supplement | Critically Low | Low |
| In‑hospital mortality of COVID-19 | >20mg/day versus never | Szarpak 2021 | Supplement | Critically Low | Very low |
| Cancer outcomes |  |  |  |  |  |
| Colorectal cancer | 5 mg/day zinc increase | Qiao 2013 | Diet | Critically Low | High |
| Esophageal cancer | 5 mg/day zinc increase | Ma 2018 | Diet | Critically Low | High |
| Digestive tract cancers | Highest versus lowest | Li 2014 | Diet | Low | High |
| Colorectal cancer | Highest versus lowest | Li 2014 | Diet | Low | Low |
| Pancreatic cancer | Highest versus lowest | Li 2017 | Diet | Critically Low | Low |
| Prostate cancer | Highest versus lowest | Mahmoud 2016 | Diet | Critically Low | High |
| Prostate cancer | 100mg/day zinc increase | Mahmoud 2016 | Diet | Critically Low | Moderate |
| Gastric cancer | Highest versus lowest | Li 2014 | Diet | Low | Very low |
| Esophageal cancer | Highest versus lowest | Ma 2018 | Diet | Critically Low | Moderate |
| Maternal and associated outcomes |  |  |  |  |  |
| Childhood wheeze | <20mg/day versus never | Beckhaus 2015 | Supplement | Critically Low | Very low |
| Stillbirth or neonatal death | <20mg/day versus never | Ota 2015 | Supplement | Low | Very low |
| Small for gestational age | >20mg/day versus never | Oh 2020 | Supplement | Low | Low |
| Pre-eclampsia/eclampsia | >20mg/day versus never | Oh 2020 | Supplement | Low | Low |
| Childhood eczema | <20mg/day versus never | Beckhaus 2015 | Supplement | Critically Low | Very low |
| High birthweight | >20mg/day versus never | Ota 2015 | Supplement | Low | Very low |
| Preterm birth | >20mg/day versus never | Oh 2020 | Supplement | Low | Low |
| Low birthweight | >20mg/day versus never | Liu 2018 | Supplement | Critically Low | Very low |
| MUAC of neonates | <20mg/day versus never | Ota 2015 | Supplement | Low | Very low |
| Neonatal sepsis | >20mg/day versus never | Ota 2015 | Supplement | Low | Very low |
| Birthweight of neonates | >20mg/day versus never | Liu 2018 | Supplement | Critically Low | Very low |
| Head circumference of neonates | >20mg/day versus never | Ota 2015 | Supplement | Low | Very low |
| Growth and development outcomes |  |  |  |  |  |
| Height gain | <20mg/day versus never | Gera 2019 | Supplement | Critically Low | Very low |
| Head circumference | <20mg/day versus never | Gera 2019 | Supplement | Critically Low | Very low |
| Height | <20mg/day versus never | Liu 2018 | Supplement | Low | Very low |
| Weight-for-length z-scores | <20mg/day versus never | Lassi 2020 | Supplement | Critically Low | Very low |
| Weight | <20mg/day versus never | Liu 2018 | Supplement | Low | Very low |
| Weight gain | <20mg/day versus never | Gera 2019 | Supplement | Critically Low | Very low |
| WAZ | <20mg/day versus never | Liu 2018 | Supplement | Low | Very low |
| Underweight | <20mg/day versus never | Gera 2019 | Supplement | Critically Low | Low |
| Stunting | <20mg/day versus never | Gera 2019 | Supplement | Critically Low | Moderate |
| Wasting | <20mg/day versus never | Gera 2019 | Supplement | Critically Low | Moderate |
| Psycho-motor development index | <20mg/day versus never | Sajedi 2020 | Supplement | Low | Very low |
| Head circumference change | <20mg/day versus never | Gera 2019 | Supplement | Critically Low | Very low |
| HAZ change | <20mg/day versus never | Gera 2019 | Supplement | Critically Low | Very low |
| Executive function | <20mg/day versus never | Warthon-Medina 2015 | Supplement | Low | Very low |
| MUAC | <20mg/day versus never | Tam 2020 | Supplement | Low | Very low |
| Hip circumference | >20mg/day versus never | Abdollahi 2020 | Supplement | Critically Low | Very low |
| Waist-to-hip ratio | <20mg/day versus never | Mayo-Wilson 2014 | Supplement | Low | Very low |
| WAZ change | <20mg/day versus never | Gera 2019 | Supplement | Critically Low | Low |
| Weight for height z-scores | <20mg/day versus never | Tam 2020 | Supplement | Low | Very low |
| MUAC change | <20mg/day versus never | Gera 2019 | Supplement | Critically Low | Moderate |
| Weight for height z-scores change | <20mg/day versus never | Gera 2019 | Supplement | Critically Low | Moderate |
| BMI change | >20mg/day versus never | Abdollahi 2020 | Supplement | Low | Very low |
| Intelligence | <20mg/day versus never | Warthon-Medina 2015 | Supplement | Low | Very low |
| HAZ | <20mg/day versus never | Tam 2020 | Supplement | Low | Very low |
| Waist-to-hip ratio | >20mg/day versus never | Abdollahi 2020 | Supplement | Critically Low | Very low |
| Waist circumference change | >20mg/day versus never | Abdollahi 2020 | Supplement | Critically Low | Very low |
| Mental development index | <20mg/day versus never | Tam 2020 | Supplement | Low | Very low |
| Body fat percentage | >20mg/day versus never | Abdollahi 2020 | Supplement | Critically Low | Very low |
| Metabolic outcomes |  |  |  |  |  |
| Total antioxidant capacity | >20mg/day versus never | Mousavi 2020 | Supplement | Critically Low | Very low |
| Glutathione | >20mg/day versus never | Mousavi 2020 | Supplement | Critically Low | Very low |
| Change of zinc concentrations | >20mg/day versus never | Furihata 2020 | Supplement | Critically Low | Very low |
| IGF-1 levels | <20mg/day versus never | Guo 2020 | Supplement | Critically Low | Very low |
| Zinc concentrations | <20mg/day versus never | Tam 2020 | Supplement | Low | Very low |
| Type 2 Diabetes Mellitus | Highest versus lowest | Fernandez-Cao 2019 | Diet | Critically Low | Very low |
| Zinc concentrations | >20mg/day versus never | Oh 2020 | Supplement | Low | Low |
| Zinc deficiency | <20mg/day versus never | Tam 2020 | Supplement | Low | Very low |
| Malondialdehyde; | >20mg/day versus never | Hosseini 2021 | Supplement | Low | Very low |
| TNF-a | >20mg/day versus never | Hosseini 2021 | Supplement | Low | Very low |
| CRP levels | >20mg/day versus never | Hosseini 2021 | Supplement | Low | Very low |
| CRP levels | <20mg/day versus never | Mousavi 2018 | Supplement | Critically Low | Very low |
| Low-density lipoprotein cholesterol | >20mg/day versus never | Ranasinghe 2015 | Supplement | Critically Low | Very low |
| Total cholesterol | >20mg/day versus never | Ranasinghe 2015 | Supplement | Critically Low | Very low |
| Triglyceride | >20mg/day versus never | Ranasinghe 2015 | Supplement | Critically Low | Very low |
| High density lipoprotein cholesterol | >20mg/day versus never | Ranasinghe 2015 | Supplement | Critically Low | Very low |
| Serum leptin levels | >20mg/day versus never | Khorshidi 2019 | Supplement | Critically Low | Very low |
| Brain-derived neurotrophic factor levels | >20mg/day versus never | Jafari 2021 | Supplement | Low | Very low |
| IL-6 levels | >20mg/day versus never | Hosseini 2021 | Supplement | Critically Low | Very low |
| Nitric oxide level | >20mg/day versus never | Mousavi 2020 | Supplement | Critically Low | Very low |
| Reproductive outcomes |  |  |  |  |  |
| Sperm motility | >20mg/day versus never | Salas-Huetos 2018 | Supplement | Critically Low | Very low |
| Clinical pregnancy rate | >20mg/day versus never | Smits 2019 | Supplement | Low | Very low |
| Sperm concentration | >20mg/day versus never | Salas-Huetos 2018 | Supplement | Critically Low | Very low |
| Sperm morphology | >20mg/day versus never | Zhao 2016 | Supplement | Critically Low | Very low |
| Sperm volume | >20mg/day versus never | Zhao 2016 | Supplement | Critically Low | Very low |
| Sperm count | >20mg/day versus never | Zhao 2016 | Supplement | Critically Low | Very low |
| Sperm viability | >20mg/day versus never | Zhao 2016 | Supplement | Critically Low | Very low |
| Respiratory outcomes |  |  |  |  |  |
| Remaining acute viral respiratory tract infection symptoms over 7 days | >20mg/day versus never | Hunter 2021 | Supplement | Moderate | Moderate |
| Pneumonia | <20mg/day versus never | Lassi 2010 | Supplement | Critically Low | High |
| Acute lower respiratory infection | <20mg/day versus never | Roth 2010 | Supplement | Critically Low | Very low |
| Pneumonia prevalence | <20mg/day versus never | Lassi 2010 | Supplement | Critically Low | Very low |
| Acute viral respiratory tract infection symptom scores in day 3 | >20mg/day versus never | Hunter 2021 | Supplement | Moderate | High |
| Mean duration of acute viral respiratory tract infection symptom | >20mg/day versus never | Hunter 2021 | Supplement | Moderate | Moderate |
| Respiratory tract infection | <20mg/day versus never | Vlieg-Boerstra 2021 | Supplement | Critically Low | Very low |
| Lower respiratory tract infection | <20mg/day versus never | Tam 2020 | Supplement | Low | Very low |
| Common cold symptom in 1st week | <20mg/day versus never | Jackson 2000 | Supplement | Critically Low | Very low |
| Average acute viral respiratory tract infection symptom | >20mg/day versus never | Hunter 2021 | Supplement | Moderate | Moderate |
| Neurologic outcomes |  |  |  |  |  |
| Depression | Highest versus lowest | Li 2017 | Diet | Low | Low |
| Depression symptom scores | <20mg/day versus never | Yosaee 2020 | Supplement | Critically Low | Low |
| Parkinson's disease | Highest versus lowest | Cheng 2015 | Diet | Critically Low | Low |
| Parkinson's disease | >20mg/day versus never | Cheng 2015 | Diet | Critically Low | Very low |
| Digestive outcomes |  |  |  |  |  |
| Diarrhea | <20mg/day versus never | Tam 2020 | Supplement | Low | Very low |
| Hyperbilirubinemia | <20mg/day versus never | Yang 2018 | Supplement | Low | Very low |
| Skeletal outcomes |  |  |  |  |  |
| Alkaline phosphatase level | >20mg/day versus never | Ceylan 2021 | Supplement | Low | Very low |
| Femoral neck bone mineral density | <20mg/day versus never | Ceylan 2021 | Supplement | Low | Very low |
| Osteocalcin levels | <20mg/day versus never | Ceylan 2021 | Supplement | Low | Very low |
| Parathyroid hormone level | <20mg/day versus never | Ceylan 2021 | Supplement | Low | Very low |
| Bone alkaline phosphatase level | <20mg/day versus never | Ceylan 2021 | Supplement | Low | Very low |
| Lumbar bone mineral density | <20mg/day versus never | Ceylan 2021 | Supplement | Low | Very low |
| Overall bone health complications | >20mg/day versus never | Ceylan 2021 | Diet | Low | Very low |
| Other outcomes |  |  |  |  |  |
| Tinnitus improvement | >20mg/day versus never | Person 2016 | Supplement | Critically Low | Very low |
| Malaria | >20mg/day versus never | Mayo-Wilson 2014 | Supplement | Low | Very low |
| At least one otitis media | >20mg/day versus never | Gulani 2012 | Supplement | Moderate | Very low |
| Anemia | >20mg/day versus never | Tam 2020 | Supplement | Low | Very low |

**Table S3. AMSTAR2 grades and GRADE classifications of zinc intakes.**

AMSTAR, assessing the methodological quality of systematic reviews; BMI, body mass index; CRP, C-reactive protein; GRADE, grading of recommendations assessment, development, and evaluation; HAZ, height-for-age z-scores; IGF-1, insulin-like growth factors -1; IL, interleukin; MUAC, mid-upper arm circumference; TNF-a, tumor necrosis factor-alpha; WAZ, weight for-age z-scores.
