# Supplementary material for: Zinc Intakes and Health Outcomes: An Umbrella Review
Source: Front Nutr. 2022 Feb 8;9:798078. doi: 10.3389/fnut.2022.798078 (PMC8861317; doi:10.3389/fnut.2022.798078)
Supplement: Supplementary file 4 [file Table_4.docx]

| Outcome | Assessed with | Author-Year | AMSTAR2 items♯ | | | | | | | | | | | | | | | | |
| --- | --- | --- | --- | --- | --- | --- | --- | --- | --- | --- | --- | --- | --- | --- | --- | --- | --- | --- | --- |
|  |  |  | 1 | 2 | 3 | 4 | 5 | 6 | 7 | 8 | 9 | 10 | 11 | 12 | 13 | 14 | 15 | 16 | Overall rating |
| Mortality outcomes |  |  |  |  |  |  |  |  |  |  |  |  |  |  |  |  |  |  |  |
| All-cause mortality | Highest versus lowest | Jayedi 2018 | Yes | Yes | No | Partial Yes | Yes | Yes | No | Partial Yes | No | No | Yes | Yes | No | Yes | No | No | Critically Low |
| All-cause mortality | <20mg/day versus never | Kanellopoulou 2021 | Yes | Partial Yes | No | Partial Yes | No | No | No | Partial Yes | Yes | No | No | Yes | Yes | Yes | No | No | Critically Low |
| All-cause mortality | <20mg/day versus never | Tam 2020 | Yes | Yes | No | Partial Yes | Yes | Yes | Yes | Yes | Yes | No | Yes | Yes | Yes | No | No | No | Low |
| Survival to hospital discharge of COVID-19 | >20mg/day versus never | Szarpak 2021 | Yes | No | No | Partial Yes | Yes | Yes | No | No | Yes | No | Yes | Yes | Yes | No | No | No | Critically Low |
| In‑hospital mortality of COVID-19 | >20mg/day versus never | Szarpak 2021 | Yes | No | No | Partial Yes | Yes | Yes | No | No | Yes | No | Yes | Yes | Yes | No | No | No | Critically Low |
| Cancer outcomes |  |  |  |  |  |  |  |  |  |  |  |  |  |  |  |  |  |  |  |
| Colorectal cancer | 5 mg/day zinc increase | Qiao 2013 | No | Partial Yes | Yes | Partial Yes | No | Yes | No | Yes | No | No | No | Yes | No | No | Yes | No | Critically Low |
| Esophageal cancer | 5 mg/day zinc increase | Ma 2018 | Yes | Yes | No | Yes | No | Yes | No | Partial Yes | No | No | Yes | No | Yes | Yes | Yes | No | Critically Low |
| Digestive tract cancers | Highest versus lowest | Li 2014 | No | Partial Yes | No | Partial Yes | No | Yes | No | Partial Yes | Partial Yes | No | Yes | Yes | Yes | Yes | Yes | No | Low |
| Colorectal cancer | Highest versus lowest | Li 2014 | No | Partial Yes | No | Partial Yes | No | Yes | No | Partial Yes | Partial Yes | No | Yes | Yes | Yes | Yes | Yes | No | Low |
| Pancreatic cancer | Highest versus lowest | Li 2017 | Yes | Partial Yes | No | Partial Yes | No | Yes | No | Partial Yes | Partial Yes | No | No | Yes | Yes | Yes | Yes | No | Critically Low |
| Prostate cancer | Highest versus lowest | Mahmoud 2016 | Yes | No | No | Partial Yes | No | Yes | Yes | Yes | No | No | Yes | No | Yes | Yes | Yes | No | Critically Low |
| Prostate cancer | 100mg/day zinc increase | Mahmoud 2016 | Yes | No | No | Partial Yes | No | Yes | Yes | Yes | No | No | Yes | No | Yes | Yes | Yes | No | Critically Low |
| Gastric cancer | Highest versus lowest | Li 2014 | No | Partial Yes | No | Partial Yes | No | Yes | No | Partial Yes | Partial Yes | No | Yes | Yes | Yes | Yes | Yes | No | Low |
| Esophageal cancer | Highest versus lowest | Ma 2018 | Yes | Yes | No | Yes | No | Yes | No | Partial Yes | No | No | Yes | No | Yes | Yes | Yes | No | Critically Low |
| Maternal and ass outcomes |  |  |  |  |  |  |  |  |  |  |  |  |  |  |  |  |  |  |  |
| Childhood wheeze | <20mg/day versus never | Beckhaus 2015 | No | Partial Yes | No | Partial Yes | Yes | Yes | No | Partial Yes | Yes | No | No | No | No | Yes | No | No | Critically Low |
| Stillbirth or neonatal death | <20mg/day versus never | Ota 2015 | Yes | Yes | No | Partial Yes | Yes | Yes | Yes | Yes | Yes | No | Yes | Yes | Yes | Yes | No | No | Low |
| Small for gestational age | >20mg/day versus never | Oh 2020 | Yes | Yes | No | Yes | Yes | Yes | Yes | Partial Yes | Yes | No | Yes | Yes | Yes | No | No | No | Low |
| Pre-eclampsia/eclampsia | >20mg/day versus never | Oh 2020 | Yes | Yes | No | Yes | Yes | Yes | Yes | Partial Yes | Yes | No | Yes | Yes | Yes | No | No | No | Low |
| Childhood eczema | <20mg/day versus never | Beckhaus 2015 | No | Partial Yes | No | Partial Yes | Yes | Yes | No | Partial Yes | Yes | No | No | No | No | Yes | No | No | Critically Low |
| High birthweight | >20mg/day versus never | Ota 2015 | Yes | Yes | No | Partial Yes | Yes | Yes | Yes | Yes | Yes | No | Yes | Yes | Yes | Yes | No | No | Low |
| Preterm birth | >20mg/day versus never | Oh 2020 | Yes | Yes | No | Yes | Yes | Yes | Yes | Partial Yes | Yes | No | Yes | Yes | Yes | No | No | No | Low |
| Low birthweight | >20mg/day versus never | Liu 2018 | Yes | Partial Yes | No | Partial Yes | Yes | Yes | No | Partial Yes | Yes | No | Yes | Yes | Yes | Yes | No | No | Critically Low |
| MUCA of neonates | <20mg/day versus never | Ota 2015 | Yes | Yes | No | Partial Yes | Yes | Yes | Yes | Yes | Yes | No | Yes | Yes | Yes | Yes | No | No | Low |
| Neonatal sepsis | >20mg/day versus never | Ota 2015 | Yes | Yes | No | Partial Yes | Yes | Yes | Yes | Yes | Yes | No | Yes | Yes | Yes | Yes | No | No | Low |
| Birthweight of neonates | >20mg/day versus never | Liu 2018 | Yes | Partial Yes | No | Partial Yes | Yes | Yes | No | Partial Yes | Yes | No | Yes | Yes | Yes | Yes | No | No | Critically Low |
| Infant head circumference | >20mg/day versus never | Ota 2015 | Yes | Yes | No | Partial Yes | Yes | Yes | Yes | Yes | Yes | No | Yes | Yes | Yes | Yes | No | No | Low |
| Growth outcomes |  |  |  |  |  |  |  |  |  |  |  |  |  |  |  |  |  |  |  |
| Height gain | <20mg/day versus never | Gera 2019 | Yes | No | No | Partial Yes | Yes | Yes | No | Yes | Yes | No | Yes | Yes | Yes | No | Yes | No | Critically Low |
| Head circumference | <20mg/day versus never | Gera 2019 | Yes | No | No | Partial Yes | Yes | Yes | No | Yes | Yes | No | Yes | Yes | Yes | No | Yes | No | Critically Low |
| Height | <20mg/day versus never | Liu 2018 | Yes | Partial Yes | No | Partial Yes | Yes | Yes | No | Partial Yes | Yes | No | Yes | Yes | Yes | Yes | Yes | No | Low |
| Weight-for-length z-scores | <20mg/day versus never | Lassi 2020 | Yes | Partial Yes | No | Partial Yes | Yes | Yes | Yes | Yes | Yes | No | No | Yes | Yes | Yes | No | No | Critically Low |
| Weight | <20mg/day versus never | Liu 2018 | Yes | Partial Yes | No | Partial Yes | Yes | Yes | No | Partial Yes | Yes | No | Yes | Yes | Yes | Yes | Yes | No | Low |
| Weight gain | <20mg/day versus never | Gera 2019 | Yes | No | No | Partial Yes | Yes | Yes | No | Yes | Yes | No | Yes | Yes | Yes | No | Yes | No | Critically Low |
| WAZ | <20mg/day versus never | Liu 2018 | Yes | Partial Yes | No | Partial Yes | Yes | Yes | No | Partial Yes | Yes | No | Yes | Yes | Yes | Yes | Yes | No | Low |
| Underweight | <20mg/day versus never | Gera 2019 | Yes | No | No | Partial Yes | Yes | Yes | No | Yes | Yes | No | Yes | Yes | Yes | No | Yes | No | Critically Low |
| Stunting | <20mg/day versus never | Gera 2019 | Yes | No | No | Partial Yes | Yes | Yes | No | Yes | Yes | No | Yes | Yes | Yes | No | Yes | No | Critically Low |
| Wasting | <20mg/day versus never | Gera 2019 | Yes | No | No | Partial Yes | Yes | Yes | No | Yes | Yes | No | Yes | Yes | Yes | No | Yes | No | Critically Low |
| Psycho-motor development index | <20mg/day versus never | Sajedi 2020 | Yes | Partial Yes | No | Yes | Yes | Yes | No | No | Yes | No | Yes | No | Yes | No | Yes | No | Low |
| Head circumference change | <20mg/day versus never | Gera 2019 | Yes | No | No | Partial Yes | Yes | Yes | No | Yes | Yes | No | Yes | Yes | Yes | No | Yes | No | Critically Low |
| HAZ change | <20mg/day versus never | Gera 2019 | Yes | No | No | Partial Yes | Yes | Yes | No | Yes | Yes | No | Yes | Yes | Yes | No | Yes | No | Critically Low |
| Executive function | <20mg/day versus never | Warthon-Medina 2015 | Yes | Partial Yes | No | Partial Yes | No | Yes | Yes | Partial Yes | Yes | No | Yes | Yes | Yes | No | No | No | Low |
| MUAC | <20mg/day versus never | Tam 2020 | Yes | Yes | No | Partial Yes | Yes | Yes | Yes | Yes | Yes | No | Yes | Yes | Yes | No | No | No | Low |
| Hip circumference | >20mg/day versus never | Abdollahi 2020 | Yes | Yes | No | Partial Yes | Yes | Yes | No | Partial Yes | Yes | No | Yes | Yes | Yes | Yes | No | No | Critically Low |
| Waist-to-hip ratio | <20mg/day versus never | Mayo-Wilson 2014 | No | Partial Yes | No | Partial Yes | Yes | Yes | Yes | Partial Yes | Yes | No | Yes | Yes | Yes | Yes | No | No | Low |
| WAZ change | <20mg/day versus never | Gera 2019 | Yes | No | No | Partial Yes | Yes | Yes | No | Yes | Yes | No | Yes | Yes | Yes | No | Yes | No | Critically Low |
| Weight for height z-scores | <20mg/day versus never | Tam 2020 | Yes | Yes | No | Partial Yes | Yes | Yes | Yes | Yes | Yes | No | Yes | Yes | Yes | No | No | No | Low |
| MUAC change | <20mg/day versus never | Gera 2019 | Yes | No | No | Partial Yes | Yes | Yes | No | Yes | Yes | No | Yes | Yes | Yes | No | Yes | No | Critically Low |
| Weight for height z-scores change | <20mg/day versus never | Gera 2019 | Yes | No | No | Partial Yes | Yes | Yes | No | Yes | Yes | No | Yes | Yes | Yes | No | Yes | No | Critically Low |
| BMI change | >20mg/day versus never | Abdollahi 2020 | Yes | Yes | No | Partial Yes | Yes | Yes | No | Partial Yes | Yes | No | Yes | Yes | Yes | Yes | Yes | No | Low |
| Intelligence | <20mg/day versus never | Warthon-Medina 2015 | Yes | Partial Yes | No | Partial Yes | No | Yes | Yes | Partial Yes | Yes | No | Yes | Yes | Yes | No | No | No | Low |
| HAZ | <20mg/day versus never | Tam 2020 | Yes | Yes | No | Partial Yes | Yes | Yes | Yes | Yes | Yes | No | Yes | Yes | Yes | No | No | No | Low |
| Waist-to-hip ratio | >20mg/day versus never | Abdollahi 2020 | Yes | Yes | No | Partial Yes | Yes | Yes | No | Partial Yes | Yes | No | Yes | Yes | Yes | Yes | No | No | Critically Low |
| Waist circumference change | >20mg/day versus never | Abdollahi 2020 | Yes | Yes | No | Partial Yes | Yes | Yes | No | Partial Yes | Yes | No | Yes | Yes | Yes | Yes | No | No | Critically Low |
| Mental development index | <20mg/day versus never | Tam 2020 | Yes | Yes | No | Partial Yes | Yes | Yes | Yes | Yes | Yes | No | Yes | Yes | Yes | No | No | No | Low |
| Body fat percentage | >20mg/day versus never | Abdollahi 2020 | Yes | Yes | No | Partial Yes | Yes | Yes | No | Partial Yes | Yes | No | Yes | Yes | Yes | Yes | No | No | Critically Low |
| Metabolic outcomes |  |  |  |  |  |  |  |  |  |  |  |  |  |  |  |  |  |  |  |
| Total antioxidant capacity | >20mg/day versus never | Mousavi 2020 | Yes | Yes | No | Partial Yes | No | Yes | No | Yes | Yes | No | No | No | No | Yes | No | No | Critically Low |
| Glutathione | >20mg/day versus never | Mousavi 2020 | Yes | Yes | No | Partial Yes | No | Yes | No | Yes | Yes | No | No | No | No | Yes | No | No | Critically Low |
| Change of zinc concentrations | >20mg/day versus never | Furihata 2020 | Yes | Yes | No | Partial Yes | Yes | No | No | Yes | Yes | No | Yes | Yes | Yes | Yes | No | No | Critically Low |
| IGF-1 levels | <20mg/day versus never | Guo 2020 | Yes | Partial Yes | No | Partial Yes | Yes | Yes | No | Partial Yes | Yes | No | Yes | Yes | No | Yes | Yes | No | Critically Low |
| Zinc concentrations | <20mg/day versus never | Tam 2020 | Yes | Yes | No | Partial Yes | Yes | Yes | Yes | Yes | Yes | No | Yes | Yes | Yes | No | No | No | Low |
| Type 2 Diabetes Mellitus | Highest versus lowest | Fernandez-Cao 2019 | Yes | Yes | No | Partial Yes | Yes | Yes | No | Yes | No | No | Yes | No | No | Yes | Yes | No | Critically Low |
| Zinc concentrations | >20mg/day versus never | Oh 2020 | Yes | Yes | No | Yes | Yes | Yes | Yes | Partial Yes | Yes | No | Yes | Yes | Yes | No | No | No | Low |
| Zinc deficiency | <20mg/day versus never | Tam 2020 | Yes | Yes | No | Partial Yes | Yes | Yes | Yes | Yes | Yes | No | Yes | Yes | Yes | No | No | No | Low |
| Malondialdehyde | >20mg/day versus never | Hosseini 2021 | Yes | Yes | No | Partial Yes | Yes | Yes | No | Yes | Yes | No | Yes | No | Yes | Yes | Yes | No | Low |
| TNF-a | >20mg/day versus never | Hosseini 2021 | Yes | Yes | No | Partial Yes | Yes | Yes | No | Yes | Yes | No | Yes | No | Yes | Yes | Yes | No | Low |
| CRP levels | >20mg/day versus never | Hosseini 2021 | Yes | Yes | No | Partial Yes | Yes | Yes | No | Yes | Yes | No | Yes | No | Yes | Yes | Yes | No | Low |
| CRP levels | <20mg/day versus never | Mousavi 2018 | Yes | Yes | No | Partial Yes | No | Yes | No | Yes | Yes | No | Yes | No | Yes | Yes | No | No | Critically Low |
| Low-density lipoprotein cholesterol | >20mg/day versus never | Ranasinghe 2015 | Yes | Partial Yes | No | Partial Yes | Yes | No | Yes | Yes | No | No | No | No | No | No | No | No | Critically Low |
| Total cholesterol | >20mg/day versus never | Ranasinghe 2015 | Yes | Partial Yes | No | Partial Yes | Yes | No | Yes | Yes | No | No | No | No | No | No | No | No | Critically Low |
| Triglyceride | >20mg/day versus never | Ranasinghe 2015 | Yes | Partial Yes | No | Partial Yes | Yes | No | Yes | Yes | No | No | No | No | No | No | No | No | Critically Low |
| High density lipoprotein cholesterol | >20mg/day versus never | Ranasinghe 2015 | Yes | Partial Yes | No | Partial Yes | Yes | No | Yes | Yes | No | No | No | No | No | No | No | No | Critically Low |
| Serum leptin levels | >20mg/day versus never | Khorshidi 2019 | Yes | Partial Yes | No | Partial Yes | No | Yes | No | Yes | No | No | Yes | No | No | Yes | Yes | No | Critically Low |
| Brain-derived neurotrophic factor levels | >20mg/day versus never | Jafari 2021 | No | Partial Yes | No | Partial Yes | Yes | Yes | No | Yes | Yes | No | Yes | Yes | Yes | Yes | Yes | No | Low |
| IL-6 levels | >20mg/day versus never | Hosseini 2021 | Yes | Yes | No | Partial Yes | Yes | Yes | No | Yes | Yes | No | Yes | No | Yes | Yes | No | No | Critically Low |
| Nitric oxide level | >20mg/day versus never | Mousavi 2020 | Yes | Yes | No | Partial Yes | No | Yes | No | Yes | Yes | No | No | No | No | Yes | No | No | Critically Low |
| Reproductive outcomes |  |  |  |  |  |  |  |  |  |  |  |  |  |  |  |  |  |  |  |
| Sperm motility | >20mg/day versus never | Salas-Huetos 2018 | Yes | Partial Yes | No | Partial Yes | Yes | Yes | No | Partial Yes | Yes | No | Yes | Yes | Yes | No | No | No | Critically Low |
| Clinical pregnancy rate | >20mg/day versus never | Smits 2019 | Yes | Yes | No | Yes | Yes | Yes | Yes | Yes | Yes | Yes | Yes | Yes | Yes | Yes | No | No | Low |
| Sperm concentration | >20mg/day versus never | Salas-Huetos 2018 | Yes | Partial Yes | No | Partial Yes | Yes | Yes | No | Partial Yes | Yes | No | Yes | Yes | Yes | No | No | No | Critically Low |
| Sperm morphology | >20mg/day versus never | Zhao 2016 | Yes | No | No | Partial Yes | Yes | Yes | No | Partial Yes | No | No | Yes | No | No | No | No | No | Critically Low |
| Sperm volume | >20mg/day versus never | Zhao 2016 | Yes | No | No | Partial Yes | Yes | Yes | No | Partial Yes | No | No | Yes | No | No | No | No | No | Critically Low |
| Sperm count | >20mg/day versus never | Zhao 2016 | Yes | No | No | Partial Yes | Yes | Yes | No | Partial Yes | No | No | Yes | No | No | No | No | No | Critically Low |
| Sperm viability | >20mg/day versus never | Zhao 2016 | Yes | No | No | Partial Yes | Yes | Yes | No | Partial Yes | No | No | Yes | No | No | No | No | No | Critically Low |
| Respiratory outcomes |  |  |  |  |  |  |  |  |  |  |  |  |  |  |  |  |  |  |  |
| Remaining acute viral respiratory tract infection symptoms over 7 days | >20mg/day versus never | Hunter 2021 | Yes | Yes | No | Partial Yes | No | Yes | Yes | Yes | Yes | No | Yes | Yes | Yes | Yes | Yes | No | Moderate |
| Pneumonia | <20mg/day versus never | Lassi 2010 | Yes | Partial Yes | No | Partial Yes | Yes | Yes | Yes | Yes | Yes | No | No | Yes | Yes | Yes | No | No | Critically Low |
| Acute lower respiratory infection | <20mg/day versus never | Roth 2010 | Yes | No | No | Partial Yes | Yes | Yes | Yes | Partial Yes | No | No | Yes | Yes | No | Yes | Yes | No | Critically Low |
| Pneumonia prevalence | <20mg/day versus never | Lassi 2010 | Yes | Partial Yes | No | Partial Yes | Yes | Yes | Yes | Yes | Yes | No | No | Yes | Yes | Yes | No | No | Critically Low |
| Acute viral respiratory tract infection symptom in day 3 | >20mg/day versus never | Hunter 2021 | Yes | Yes | No | Partial Yes | No | Yes | Yes | Yes | Yes | No | Yes | Yes | Yes | Yes | Yes | No | Moderate |
| Mean duration of acute viral respiratory tract infection symptom | >20mg/day versus never | Hunter 2021 | Yes | Yes | No | Partial Yes | No | Yes | Yes | Yes | Yes | No | Yes | Yes | Yes | Yes | Yes | No | Moderate |
| Respiratory tract infection | <20mg/day versus never | Vlieg-Boerstra 2021 | Yes | No | No | Partial Yes | Yes | Yes | No | Yes | Yes | No | Yes | Yes | Yes | No | No | No | Critically Low |
| Lower respiratory tract infection | <20mg/day versus never | Tam 2020 | Yes | Yes | No | Partial Yes | Yes | Yes | Yes | Yes | Yes | No | Yes | Yes | Yes | No | No | No | Low |
| Common cold symptom in 1st week | <20mg/day versus never | Jackson 2000 | No | Yes | No | No | No | No | Yes | No | No | No | Yes | No | Yes | No | Yes | No | Critically Low |
| Average acute viral respiratory tract infection symptom | >20mg/day versus never | Hunter 2021 | Yes | Yes | No | Partial Yes | No | Yes | Yes | Yes | Yes | No | Yes | Yes | Yes | Yes | Yes | No | Moderate |
| Neurologic outcomes |  |  |  |  |  |  |  |  |  |  |  |  |  |  |  |  |  |  |  |
| Depression | Highest versus lowest | Li 2017 | Yes | Partial Yes | No | Partial Yes | Yes | Yes | No | Partial Yes | Partial Yes | No | Yes | No | Yes | Yes | Yes | No | Low |
| Depression symptom scores | <20mg/day versus never | Yosaee 2020 | Yes | Yes | No | Partial Yes | Yes | Yes | No | Partial Yes | No | No | Yes | No | No | Yes | Yes | No | Critically Low |
| Parkinson's disease | Highest versus lowest | Cheng 2015 | Yes | Partial Yes | No | Partial Yes | Yes | Yes | No | Partial Yes | No | No | Yes | Yes | No | No | No | No | Critically Low |
| Parkinson's disease | >20mg/day versus never | Cheng 2015 | Yes | Partial Yes | No | Partial Yes | Yes | Yes | No | Partial Yes | No | No | Yes | Yes | No | No | No | No | Critically Low |
| Digestive outcomes |  |  |  |  |  |  |  |  |  |  |  |  |  |  |  |  |  |  |  |
| Diarrhea | <20mg/day versus never | Tam 2020 | Yes | Yes | No | Partial Yes | Yes | Yes | Yes | Yes | Yes | No | Yes | Yes | Yes | No | No | No | Low |
| Hyperbilirubinemia | <20mg/day versus never | Yang 2018 | Yes | Yes | No | Partial Yes | Yes | Yes | No | Partial Yes | Yes | No | Yes | Yes | Yes | Yes | Yes | No | Low |
| Skeletal outcomes |  |  |  |  |  |  |  |  |  |  |  |  |  |  |  |  |  |  |  |
| Alkaline phosphatase level | >20mg/day versus never | Ceylan 2021 | No | Partial Yes | No | Partial Yes | No | No | No | Partial Yes | Yes | No | Yes | Yes | Yes | Yes | Yes | No | Low |
| Femoral neck bone mineral density | <20mg/day versus never | Ceylan 2021 | No | Partial Yes | No | Partial Yes | No | No | No | Partial Yes | Yes | No | Yes | Yes | Yes | Yes | Yes | No | Low |
| Osteocalcin levels | <20mg/day versus never | Ceylan 2021 | No | Partial Yes | No | Partial Yes | No | No | No | Partial Yes | Yes | No | Yes | Yes | Yes | Yes | Yes | No | Low |
| Parathyroid hormone level | <20mg/day versus never | Ceylan 2021 | No | Partial Yes | No | Partial Yes | No | No | No | Partial Yes | Yes | No | Yes | Yes | Yes | Yes | Yes | No | Low |
| Bone alkaline phosphatase level | <20mg/day versus never | Ceylan 2021 | No | Partial Yes | No | Partial Yes | No | No | No | Partial Yes | Yes | No | Yes | Yes | Yes | Yes | Yes | No | Low |
| Lumbar bone mineral density | <20mg/day versus never | Ceylan 2021 | No | Partial Yes | No | Partial Yes | No | No | No | Partial Yes | Yes | No | Yes | Yes | Yes | Yes | Yes | No | Low |
| Overall bone health complications | >20mg/day versus never | Ceylan 2021 | No | Partial Yes | No | Partial Yes | No | No | No | Partial Yes | Yes | No | Yes | Yes | Yes | Yes | Yes | No | Low |
| Other outcomes |  |  |  |  |  |  |  |  |  |  |  |  |  |  |  |  |  |  |  |
| Tinnitus improvement | >20mg/day versus never | Person 2016 | Yes | Partial Yes | No | Partial Yes | Yes | Yes | Yes | Yes | Yes | Yes | No | Yes | Yes | No | No | No | Critically Low |
| Malaria | >20mg/day versus never | Mayo-Wilson 2014 | No | Partial Yes | No | Partial Yes | Yes | Yes | Yes | Partial Yes | Yes | No | Yes | Yes | Yes | Yes | No | No | Low |
| At least one otitis media | >20mg/day versus never | Gulani 2012 | Yes | Partial Yes | No | Yes | Yes | Yes | Yes | Yes | Yes | Yes | Yes | Yes | Yes | Yes | Yes | No | Moderate |
| Anemia | >20mg/day versus never | Tam 2020 | Yes | Yes | No | Partial Yes | Yes | Yes | Yes | Yes | Yes | No | Yes | Yes | Yes | No | No | No | Low |

**Table S4. AMSTAR2 grades of the studies related to zinc intakes.**

AMSTAR, assessing the methodological quality of systematic reviews; BMI, body mass index; CRP, C-reactive protein; HAZ, height-for-age z-scores; IGF-1, insulin-like growth factors -1; IL, interleukin; MUAC, mid-upper arm circumference; TNF-a, tumor necrosis factor-alpha; WAZ, weight for-age z-scores.

♯ AMSTAR2 items include:

1. Did the research questions and inclusion criteria for the review include the components of PICO (Population, Intervention, Comparator group, Outcome)? YES/NO.

2. (critical item) Did the report of the review contain an explicit statement that the review methods were established prior to the conduct of the review and did the report justify any significant deviations from the protocol? YES, PARTIAL YES, NO.

3. Did the review authors explain their selection of the study designs for inclusion in the review? YES/NO.

4. (critical item) Did the review authors use a comprehensive literature search strategy? YES, PARTIAL YES, NO.

5. Did the review authors perform study selection in duplicate? YES/NO.

6. Did the review authors perform data extraction in duplicate? YES/NO.

7. (critical item) Did the review authors provide a list of excluded studies to justify the exclusions? YES, PARTIAL YES, NO.

8. Did the review authors describe the included studies in adequate detail? YES, PARTIAL YES, NO.

9. (critical item) Did the review authors use a satisfactory technique for assessing the risk of bias in individual studies that were included in the review? For RCTs (Randomized controlled studies): YES, PARTIAL YES, NO, INCLUDES ONLY NRSI (Non-Randomized Studies of Intervention)/ RCTs.

10. Did the review authors report on the sources of funding for the studies included in the review? YES/NO.

11. (critical item) If meta-analysis was performed, did the review authors use appropriate methods for statistical combination of results? For RCTs: YES, NO, NO META- ANALYSIS (CONDUCTED).

12. If meta-analysis was performed, did the review authors assess the potential impact of the risk of bias in individual studies on the results of the meta-analysis or other evidence synthesis? YES, NO, NO META-ANALYSIS INCLUDED.

13. (critical item) Did the review authors account for the risk of bias in individual studies when interpreting/discussing the results of the review? YES/NO.

14. Did the review authors provide a satisfactory explanation for, and discussion of, any heterogeneity observed in the results of the review? YES/NO.

15. (critical item) If they performed quantitative synthesis did the review authors carry out an adequate investigation of publication bias (small study bias) and discuss its likely impact on the results of the review? YES, NO, NO META-ANALYSIS CONDUCTED.

16. Did the review authors report any potential sources of conflict of interest, including any funding they received for conducting the review? YES/NO.
